# A transverse isotropic viscoelastic constitutive model for aortic valve tissue

**DOI:** 10.1098/rsos.160585

**Published:** 2017-01-11

**Authors:** Afshin Anssari-Benam, Andrea Bucchi, Hazel R. C. Screen, Sam L. Evans

**Affiliations:** 1 The BIONEER centre, Cardiovascular Engineering Research Laboratory (CERL), School of Engineering, University of Portsmouth, Anglesea Road, Portsmouth PO1 3DJ, UK; 2 Institute of Bioengineering, School of Engineering and Materials Science, Queen MaryUniversity of London, Mile End Road, London E1 4NS, UK; 3 School of Engineering, Cardiff University, The Parade, Cardiff CF24 3AA, UK

**Keywords:** aortic valve, transverse isotropy, rate dependency, viscoelastic model, uniaxial data, physiological rate

## Abstract

A new anisotropic viscoelastic model is developed for application to the aortic valve (AV). The directional dependency in the mechanical properties of the valve, arising from the predominantly circumferential alignment of collagen fibres, is accounted for in the form of transverse isotropy. The rate dependency of the valve's mechanical behaviour is considered to stem from the viscous (*η*) dissipative effects of the AV matrix, and is incorporated as an explicit function of the deformation rate ($\dot{\lambda }$). Model (material) parameters were determined from uniaxial tensile deformation tests of porcine AV specimens at various deformation rates, by fitting the model to each experimental dataset. It is shown that the model provides an excellent fit to the experimental data across all different rates and satisfies the condition of strict local convexity. Based on the fitting results, a nonlinear relationship between *η* and $\dot{\lambda }$ is established, highlighting a ‘shear-thinning’ behaviour for the AV with increase in the deformation rate. Using the model and these outcomes, the stress–deformation curves of the AV tissue under physiological deformation rates in both the circumferential and radial directions are predicted and presented. To verify the predictive capabilities of the model, the stress–deformation curves of AV specimens at an intermediate deformation rate were estimated and validated against the experimental data at that rate, showing an excellent agreement. While the model is primarily developed for application to the AV, it may be applied without the loss of generality to other collagenous soft tissues possessing a similar structure, with a single preferred direction of embedded collagen fibres.

## 1. Introduction

**Figure 1.**
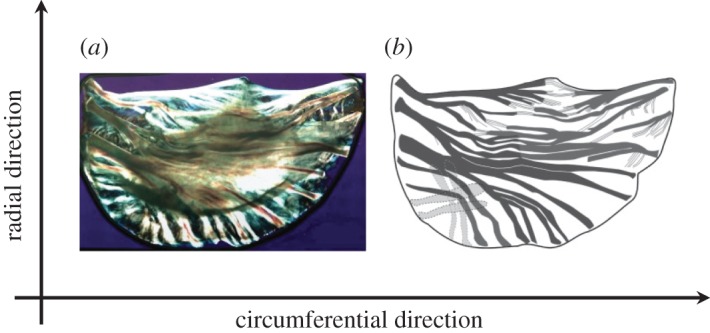
Polarized light image of an AV leaflet (*a*) and a schematic depicting the embedded fibre bundles within the valve (*b*). The principal loading directions, circumferential and radial, are indicated in the figure. The fibre structure assumes a preferred direction along the circumferential axis (adapted from [3]).

The prevalent structural component of aortic valve (AV) tissue is collagen. It comprises approximately 55% of an intact AV leaflet by dry weight [1], and is present within the tissue in the form of a network of fibres. The fibres are embedded within a viscous ‘gel-like’ matrix of glycosaminoglycans (GAGs) [2], assuming a preferred direction along the circumferential axis of each leaflet, as shown in figure 1. This preferred alignment of the collagen fibres along the circumferential direction endows the AV with strong directional dependency in its mechanical behaviour and material properties; uniaxial and biaxial tensile tests have demonstrated a significant distinction in the elastic properties of the tissue in the circumferential compared with the transverse (radial) direction [4–6], whereas distinctions also exist in the load-bearing capacity and the distensibility of the AV tissue in these two loading directions [5–7].

The viscoelastic characteristics of AV tissue have also been well documented. Tensile deformation tests on AV tissue specimens under various deformation rates have demonstrated a marked rate dependency of the ensuing stress–strain curves [6]. Moreover, studies investigating the time-dependent behaviour of the AV have reported stress relaxation under both uniaxial and biaxial tests [8,9], and creep under uniaxial conditions [9–11].

Based on the abovementioned attributes, the mechanical behaviour of AV tissue may be broadly classified as ‘anisotropic viscoelastic’, to reflect both directional and rate dependency of the mechanical properties of the tissue. Therefore, for mathematical continuum-based models to adequately and appropriately characterize the mechanical behaviour of the AV, it is imperative that both directional- and rate-dependency features are suitably incorporated and accounted for in such models. However, most mathematical continuum-based AV models developed to date have been derived under the assumption of hyperelasticity [7,12–15], and hence, in spite of providing a good fit to experimental stress–strain data obtained at any specific deformation rate, such models cannot, by definition, account for rate effects, nor model AV mechanics over a range of rate-dependent loading conditions. While discounting the rate effects may not produce significant discrepancies between experimental data and model predictions at lower deformation rates, as achievable in typical experiments, the strain rate experienced by the native AV *in vivo* is in the range of 15 000%** **min^−1^ [16]. Such high rates are not achievable by conventional material testing devices *in vitro*, and are likely to affect the material properties and the behaviour of the tissue. Therefore, models that incorporate deformation rate effects are required for accurate description of the *in vivo* mechanical behaviour of the AV.

In addition to discounting the rate effects, most of the currently developed continuum-based models also pose theoretical limitations and misperceptions in the way they incorporate directional dependency into the mechanical behaviour of the AV. To characterize a suitable class of anisotropy for the AV, an appropriate set of experimental stress–strain data (where the components of stress or strain can be independently controlled from one another) is needed. Because the thickness of the AV is much smaller than the other two in-plane dimensions (by two orders of magnitude), the AV is considered as a planar tissue [7,17]. This inherent geometrical characteristic of the AV implies that, from an experimental point of view, it would be very difficult, if not entirely impractical, to achieve stress–deformation data along the third dimension of the tissue, i.e. through its thickness, hence one is confined to only the in-plane dataset. Because the currently available uniaxial and biaxial material testing machines do not allow the independent control of in-plane shear from tensile deformation, both biaxial and uniaxial tensile tests only facilitate two components of strain/stress to be independently varied, which in turn will only sanction characterization of two independent components of the strain energy function *W*, in the form of two partial derivatives of *W* with respect to strain invariants (∂*W*/∂*I*). A rigorous analysis on this point, and the extent of suitability of the in-plane datasets, has been carried out and presented by Ogden & Holzapfel [18,19]. In this specific context, biaxial loading conditions, while more closely resembling the in-plane deformation experienced by the AV *in vivo*, do not offer any specific advantages over uniaxial datasets, which can also deliver stress–strain data on each of the two principal loading directions and provide enough data to characterize two independent ∂*W*/∂*I* terms [20,21]. However, whether the experimental datasets are achieved through uniaxial or biaxial tests, characterization of two independent ∂*W*/∂*I* terms will at best permit formulating a ‘transversely isotropic’ behaviour for inclusion into a mathematical continuum-based model [18–21]. This important point has often been overlooked in the literature and requires revisiting in formulating a new mathematical model for AV tissue. We further note that transverse isotropy is also structurally motivated in the case of AV tissue, as collagen fibres primarily assume a preferred direction along the circumferential axis (figure 1).

In this study, we derive a transversely isotropic viscoelastic model for application to the AV, incorporating the deformation rate as an explicit variable. The considered rate effects are reflected in the form of viscous damping *η* and are motivated by the dissipative effects of the valve's matrix, encompassing the viscous-like behaviour of GAGs and fibre kinematics. We start by demonstrating the general three-dimensional theory, and then apply it to uniaxial loading. Uniaxial tests were performed in circumferential and radial directions, under various deformation rates, to allow characterization of rate dependency as well as anisotropy. Appropriate mathematical constraints to comply with the condition of convexity are derived and verified against, to ensure appropriate parameter estimation. Based on the modelling results, a nonlinear relationship between the viscous damping effects and the deformation rate $\dot{\lambda }$ is established, characterizing a ‘shear-thinning’ behaviour for the AV tissue. Using this relationship, the viscous effects and subsequently the stress–deformation curves of the AV at a physiological deformation rate in both circumferential and radial directions are predicted and presented.

## 2. Continuum mechanics framework

### 2.1. Preliminaries

Following Pioletti *et al*. [22], the second Piola–Kirchhoff stress tensor **S** for a viscoelastic material undergoing large deformations, with strain rate as an explicit variable, may be expressed as

2.1 $$\text{S}=\text{S}(\text{C},\dot{\text{C}}),$$

where **C** is the right Cauchy–Green tensor, which is related to the deformation gradient tensor **F** by

2.2 $$\text{C}={\text{F}}^{T}\cdot \text{F}.$$

We note that $\dot{\text{C}}$ is the time derivative of **C**.

In the presence of viscous effects, the stress tensor **S** in equation (2.1) may be derived [22] as

2.3 $$\text{S}(\text{C},\dot{\text{C}})=2\frac{\partial W_{e}}{\partial \text{C}}+2\frac{\partial W_{v}}{\partial \dot{\text{C}}},$$

where *W*_e_ and *W*_v_ are referred to as the elastic strain and the viscous dissipation energy functions, respectively.

For an incompressible material, equation (2.3) is replaced by

2.4 $$\text{S}(\text{C},\dot{\text{C}})=2\frac{\partial W_{e}}{\partial \text{C}}-p{\text{C}}^{-1}+2\frac{\partial W_{v}}{\partial \dot{\text{C}}},$$

where *p* is the arbitrary Lagrange multiplier, enforcing the constraint of incompressibility.

### 2.2. Material symmetry

Collagen fibres are the main load-bearing component of the AV extracellular matrix, providing reinforcement to the tissue. The fibres are embedded within a gel-like viscous ground substance formed of GAGs. This structure renders the AV tissue analogous to fibre composite materials [2]. The mean fibre orientation within this composite is identified by a single direction [3,23], referred to as the ‘circumferential’ direction. The transverse direction is referred to as the ‘radial’ direction. Together, these two directions are known as the principal loading directions of the AV, shown in figure 1 in relation to a single valve leaflet. This single preferred fibre direction endows the valve with pronounced directional-dependent mechanical properties between the two principal transverse directions, i.e. transverse isotropy. Accordingly, for formulating an appropriate continuum-based model, we shall specialize the class of anisotropy to transverse isotropy, with the preferred direction of the fibres aligned along the circumferential direction. We note that AV tissue is morphologically composed of three layers, namely the fibrosa, spongiosa and ventricularis, where collagen fibres are localized within the fibrosa and ventricularis layers. While the distribution of collagen fibre orientation within those layers reflects a certain degree of dispersity, small angle light scattering studies have established that the mean preferred fibre direction within the AV tissue is predominantly along the circumferential direction [14,23]. Therefore, in our approach, we treat the tissue macroscopically as a monolayer, with the global preferred direction of fibres along the circumferential loading direction of the tissue. For a detailed analysis on how to incorporate fibre orientation dispersion into mathematical models, the interested reader may wish to read the contributions made by Freed *et al*. [24], Gasser *et al*. [25] and Holzapfel *et al*. [26].

### 2.3. Energy functions *W*

Elastic and viscous potential functions appearing in equation (2.4) may be described by (**C**) and $\left(\text{C},\dot{\text{C}}\right),$ respectively [27]:

2.5 $$\begin{array}{rl} W_{e} & =W_{e}(\text{C})\\ \text{and}\;W_{v} & =W_{v}(\text{C},\dot{\text{C}}). \end{array}\}$$

In the case of transverse isotropy, *W*_e_ may be expressed as a function of 5 invariants [18], and *W*_v_ as a function of 17 invariants [28]:

2.6 $$\begin{array}{rl} W_{e} & =W_{e}(I_{1},I_{2},I_{3},I_{4},I_{5})\\ \text{and}\;W_{v} & =W_{v}(I_{1},I_{2},I_{3},I_{4},I_{5},J_{1},J_{2},J_{3},J_{4},J_{5},J_{6},J_{7},J_{8},J_{9},J_{10},J_{11},J_{12}), \end{array}\}$$

where

2.7 $$\begin{array}{rl} I_{1} & =\mathrm{tr}\;\left(\text{C}\right),I_{2}=\frac{1}{2}[{\left(\mathrm{tr}\;\text{C}\right)}^{2}-\mathrm{tr}({\text{C}}^{2})],I_{3}=\det(\text{C}),\\ I_{4} & =\text{M}\cdot (\text{CM})\;\text{and}\;I_{5}=\text{M}\cdot ({\text{C}}^{2}\text{M}), \end{array}\}$$

and *J*_1_–*J*_12_ are the invariants of $\dot{\text{C}}$ defined as follows:

2.8 $$\begin{array}{rl} J_{1} & =\mathrm{tr}(\dot{\text{C}}),J_{2}=\mathrm{tr}\;{\left(\dot{\text{C}}\right)}^{2},J_{3}=\det(\dot{\text{C}}),J_{4}=\text{M}\cdot (\dot{\text{C}}\text{M}),J_{5}=\text{M}\cdot ({\dot{\text{C}}}^{2}\text{M}),\\ J_{6} & =\mathrm{tr}(\text{C}\dot{\text{C}}),J_{7}=\mathrm{tr}(\text{C}{\dot{\text{C}}}^{2}),J_{8}=\mathrm{tr}({\text{C}}^{2}\dot{\text{C}}),J_{9}=\mathrm{tr}({\text{C}}^{2}{\dot{\text{C}}}^{2}),J_{10}=\mathrm{tr}(\text{MC}\dot{\text{C}}\text{M}),\\ \text{and}\;J_{11} & =\mathrm{tr}(\text{MC}{\dot{\text{C}}}^{2}\text{M})\;\text{and}\;J_{12}=\mathrm{tr}(\text{M}{\text{C}}^{2}\dot{\text{C}}\text{M}). \end{array}\}$$

**Figure 2.**
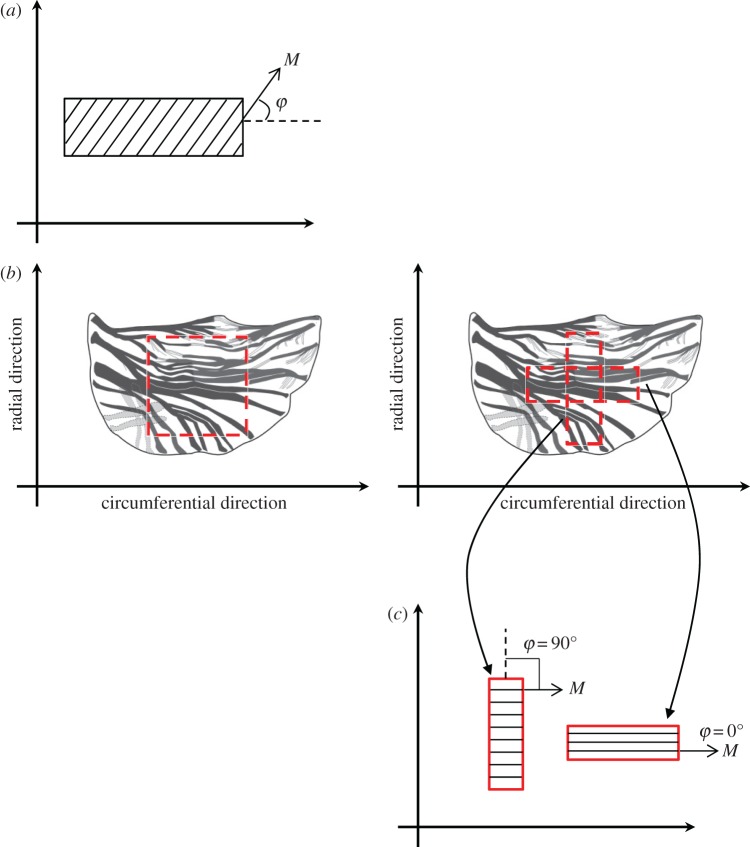
(*a*) Preferred fibre direction *M* and the angle of the family of the fibres *φ*; (*b*) square or rectangular specimens are prepared from the central region of the AV leaflet for biaxial or uniaxial tensile tests, respectively. Note that, for uniaxial tests, samples are obtained from both circumferential and radial directions; (*c*) for circumferentially cut samples, the fibre family is aligned with the principal direction. For radially cut samples, the fibre family makes an angle of 90° with the principal direction.

**M** in equations (2.7) and (2.8) denotes the preferred fibre direction given by **M **=** **[cos** ***φ*,sin** ***φ*,0]^T^ and is depicted schematically in figure 2*a*.

### 2.4. Viscoelastic stress tensors **S** and *σ*

The second Piola–Kirchhoff stress tensor **S** in equation (2.4) may be rewritten as

2.9 $$\text{S}(\text{C},\dot{\text{C}})=2\frac{\partial W_{e}}{\partial I_{i}}\cdot \frac{\partial I_{i}}{\partial \text{C}}-p{\text{C}}^{-1}+2\frac{\partial W_{v}}{\partial J_{i}}\cdot \frac{\partial J_{i}}{\partial \dot{\text{C}}}.$$

We note that, from matrix calculus, $\partial I_{i}\text{/}\partial \dot{\text{C}}=0,i=1,\ldots ,5.$

In order to develop equation (2.9) further, one needs to establish the expressions for ∂*I_i_*/∂**C** and $\partial J_{i}\text{/}\partial \dot{\text{C}},$ as follows

2.10 $$\begin{array}{rl} \frac{\partial I_{i}}{\partial \text{C}} & \equiv \begin{array}{rl} \frac{\partial I_{1}}{\partial \text{C}} & =\frac{\partial \mathrm{tr}\;\text{C}}{\partial \text{C}}=\text{I},\;\frac{\partial I_{2}}{\partial \text{C}}=\mathrm{tr}\;\text{CI}-{\text{C}}^{T},\;\frac{\partial I_{3}}{\partial \text{C}}=\det(\text{C}){\left[{\text{C}}^{-1}\right]}^{T}=I_{3}{\left[{\text{C}}^{-1}\right]}^{T},\\ \frac{\partial I_{4}}{\partial \text{C}} & =\text{M}⊗\text{M}\;\text{and}\;\frac{\partial I_{5}}{\partial \text{C}}=\text{CM}⊗\text{M}+\text{M}⊗\text{MC}, \end{array}\}\\ \frac{\partial J_{i}}{\partial \dot{\text{C}}} & \equiv \begin{array}{rl} \frac{\partial J_{1}}{\partial \dot{\text{C}}} & =\frac{\partial \mathrm{tr}\;\dot{\text{C}}}{\partial \dot{\text{C}}}=\text{I},\;\frac{\partial J_{2}}{\partial \dot{\text{C}}}=\frac{\partial \mathrm{tr}\;{\dot{\text{C}}}^{2}}{\partial \dot{\text{C}}}=2{\left[\dot{\text{C}}\right]}^{T},\;\frac{\partial J_{3}}{\partial \dot{\text{C}}}=\frac{\partial \det\dot{\text{C}}}{\partial \dot{\text{C}}}=\det\dot{\text{C}}{\left[{\dot{\text{C}}}^{-1}\right]}^{T}=J_{3}{\left[{\dot{\text{C}}}^{-1}\right]}^{T}\\ \frac{\partial J_{4}}{\partial \dot{\text{C}}} & =\text{M}⊗\text{M},\;\frac{\partial J_{5}}{\partial \dot{\text{C}}}=\dot{\text{C}}\text{M}⊗\text{M}+\text{M}⊗\text{M}\dot{\text{C}},\;\frac{\partial J_{6}}{\partial \dot{\text{C}}}={\text{C}}^{T},\;\frac{\partial J_{7}}{\partial \dot{\text{C}}}={\left[\text{C}\dot{\text{C}}\right]}^{T}+{\left[\dot{\text{C}}\text{C}\right]}^{T},\\ \frac{\partial J_{8}}{\partial \dot{\text{C}}} & ={\left[{\text{C}}^{2}\right]}^{T},\;\frac{\partial J_{9}}{\partial \dot{\text{C}}}={\left[{\text{C}}^{2}\dot{\text{C}}\right]}^{T}+{\left[\dot{\text{C}}{\text{C}}^{2}\right]}^{T},\;\frac{\partial J_{10}}{\partial \dot{\text{C}}}={\left[\text{CM}⊗\text{M}\right]}^{T},\\ \frac{\partial J_{11}}{\partial \dot{\text{C}}} & =\text{CM}⊗\text{M}\dot{\text{C}}+\dot{\text{CM}}⊗\text{MC}\;\text{and}\;\frac{\partial J_{12}}{\partial \dot{\text{C}}}={\left[{\text{C}}^{2}\text{M}⊗\text{M}\right]}^{T}, \end{array}\} \end{array}$$

where **I** denotes the identity matrix and ⊗ is the tensor product.

Substituting the expressions from equation (2.10) into equation (2.9) yields

2.11 $$\begin{array}{rl} \text{S} & =-p{\text{C}}^{-1}+2{\left(W_{e}\right)}_{1}\text{I}+2{\left(W_{e}\right)}_{2}(\text{tr}\;\text{C}\text{I}-{\text{C}}^{T})+2{\left(W_{e}\right)}_{3}(I_{3}{\left[{\text{C}}^{-1}\right]}^{T})+2{\left(W_{e}\right)}_{4}(\text{M}⊗\text{M})\\ & \;+2{\left(W_{e}\right)}_{5}(\text{CM}⊗\text{M}+\text{M}⊗\text{MC})+2{\left(W_{v}\right)}_{1}\text{I}+4{\left(W_{v}\right)}_{2}{\left[\dot{\text{C}}\right]}^{T}+2{\left(W_{v}\right)}_{3}(J_{3}{\left[{\dot{\text{C}}}^{-1}\right]}^{T})\\ & \;+2{\left(W_{v}\right)}_{4}(\text{M}⊗\text{M})+2{\left(W_{v}\right)}_{5}(\dot{\text{C}}\text{M}⊗\text{M}+\text{M}⊗\text{M}\dot{\text{C}})+2{\left(W_{v}\right)}_{6}{\left[\text{C}\right]}^{T}\\ & \;+2{\left(W_{v}\right)}_{7}({\left[\text{C}\dot{\text{C}}\right]}^{T}+{\left[\dot{\text{C}}\text{C}\right]}^{T})+2{\left(W_{v}\right)}_{8}{\left[{\text{C}}^{2}\right]}^{T}+2{\left(W_{v}\right)}_{9}({\left[{\text{C}}^{2}\dot{\text{C}}\right]}^{T}+{\left[\dot{\text{C}}{\text{C}}^{2}\right]}^{T})\\ & \;+2{\left(W_{v}\right)}_{10}{\left[\text{CM}⊗\text{M}\right]}^{T}+2{\left(W_{v}\right)}_{11}(\text{CM}⊗\text{M}\dot{\text{C}}+\dot{\text{C}}\text{M}⊗\text{MC})+2{\left(W_{v}\right)}_{12}{\left[{\text{C}}^{2}\text{M}⊗\text{M}\right]}^{T}, \end{array}$$

where, for simplicity, notations (*W*_e_)*_i_* and (*W*_v_)*_i_* have been adopted to represent ∂*W*_e_/∂*I_i_* and ∂*W*_v_/∂*J_i_*, respectively.

Under the assumption of incompressibility, *I*_3 _= det **C**=1, and therefore, equation (2.11) can be slightly simplified to

2.12 $$\begin{array}{rl} \text{S} & =-p{\text{C}}^{-1}+2{\left(W_{e}\right)}_{1}\text{I}+2{\left(W_{e}\right)}_{2}(\text{tr}\;\text{C}\text{I}-{\text{C}}^{T})+2{\left(W_{e}\right)}_{4}(\text{M}⊗\text{M})+2{\left(W_{e}\right)}_{5}(\text{CM}⊗\text{M}+\text{M}⊗\text{MC})\\ & \;+2{\left(W_{v}\right)}_{1}\text{I}+4{\left(W_{v}\right)}_{2}{\left[\dot{\text{C}}\right]}^{T}+2{\left(W_{v}\right)}_{3}(J_{3}{\left[{\dot{\text{C}}}^{-1}\right]}^{T})+2{\left(W_{v}\right)}_{4}(\text{M}⊗\text{M})\\ & \;+2{\left(W_{v}\right)}_{5}(\dot{\text{C}}\text{M}⊗\text{M}+\text{M}⊗\text{M}\dot{\text{C}})+2{\left(W_{v}\right)}_{6}{\left[\text{C}\right]}^{T}+2{\left(W_{v}\right)}_{7}({\left[\text{C}\dot{\text{C}}\right]}^{T}+{\left[\dot{\text{C}}\text{C}\right]}^{T})\\ & \;+2{\left(W_{v}\right)}_{8}{\left[{\text{C}}^{2}\right]}^{T}+2{\left(W_{v}\right)}_{9}({\left[{\text{C}}^{2}\dot{\text{C}}\right]}^{T}+{\left[\dot{\text{C}}{\text{C}}^{2}\right]}^{T})+2{\left(W_{v}\right)}_{10}{\left[\text{CM}⊗\text{M}\right]}^{T}\\ & \;+2{\left(W_{v}\right)}_{11}(\text{CM}⊗\text{M}\dot{\text{C}}+\dot{\text{C}}\text{M}⊗\text{MC})+2{\left(W_{v}\right)}_{12}{\left[{\text{C}}^{2}\text{M}⊗\text{M}\right]}^{T}. \end{array}$$

The Cauchy stress **σ**, also known as the true stress, is obtained from **σ** = **FSF**^T^, and, in view of equation (2.12), may be expressed as

2.13 $$\begin{array}{rl} \sigma & =\text{F}\{2{\left(W_{e}\right)}_{1}\text{I}+2{\left(W_{e}\right)}_{2}(\text{tr}\;\text{C}\text{I}-{\text{C}}^{T})+2{\left(W_{e}\right)}_{4}(\text{M}⊗\text{M})+2{\left(W_{e}\right)}_{5}(\text{CM}⊗\text{M}+\text{M}⊗\text{MC})\\ & \;+2{\left(W_{v}\right)}_{1}\text{I}+4{\left(W_{v}\right)}_{2}{\left[\dot{\text{C}}\right]}^{T}+2{\left(W_{v}\right)}_{3}(J_{3}{\left[{\dot{\text{C}}}^{-1}\right]}^{T})+2{\left(W_{v}\right)}_{4}(\text{M}⊗\text{M})\\ & \;+2{\left(W_{v}\right)}_{5}(\dot{\text{C}}\text{M}⊗\text{M}+\text{M}⊗\text{M}\dot{\text{C}})+2{\left(W_{v}\right)}_{6}{\left[\text{C}\right]}^{T}+2{\left(W_{v}\right)}_{7}({\left[\text{C}\dot{\text{C}}\right]}^{T}+{\left[\dot{\text{C}}\text{C}\right]}^{T})\\ & \;+2{\left(W_{v}\right)}_{8}{\left[{\text{C}}^{2}\right]}^{T}+2{\left(W_{v}\right)}_{9}({\left[{\text{C}}^{2}\dot{\text{C}}\right]}^{T}+{\left[\dot{\text{C}}{\text{C}}^{2}\right]}^{T})+2{\left(W_{v}\right)}_{10}{\left[\text{CM}⊗\text{M}\right]}^{T}\\ & \;+2{\left(W_{v}\right)}_{11}(\text{CM}⊗\text{M}\dot{\text{C}}+\dot{\text{C}}\text{M}⊗\text{MC})+2{\left(W_{v}\right)}_{12}{\left[{\text{C}}^{2}\text{M}⊗\text{M}\right]}^{T}\}{\text{F}}^{T}-p\text{I}. \end{array}$$

Equations (2.12) and (2.13) present the generic relationships for the second Piola–Kirchhoff stress tensor **S** and the Cauchy stress tensor **σ**, respectively, as a function of the right Cauchy–Green tensor **C** for a transversely isotropic incompressible viscoelastic continuum, based on equation (2.4). It must be noted that equation (2.13) contains similar expressions to that provided by Ogden for transversely isotropic elastic materials, formulated in relation to the left Cauchy–Green tensor **B** [18].

In the following, we shall consider appropriate assumptions and conditions that best describe the deformation of the AV specimens in mechanical tensile tests, in order to specialize equation (2.13) for suitable application to experimental data and estimation of the material parameters.

### 2.5. Pure homogenous deformation

When the principal axes of deformation coincide with the Cartesian coordinate directions, and the principal stretches *λ*_1_, *λ*_2_ and *λ*_3_ are independent of the coordinates (say *x*, *y* and *z*), the deformation is said to be a pure homogeneous deformation [18]. This is often the case in biaxial and uniaxial tensile deformation tests of the AV, as the specimens are prepared such that the circumferential and radial directions are often in line with the *x-* and *y*-directions of the Cartesian coordinate system. In this case, the components of the deformation gradient tensor **F** have a diagonal form diag[*λ*_1_,*λ*_2_,*λ*_3_]. It therefore follows

2.14 $$\text{F}=\left[\begin{array}{ccc} \lambda_{1} & 0 & 0\\ 0 & \lambda_{2} & 0\\ 0 & 0 & \lambda_{3} \end{array}\right],\;\text{C}=\left[\begin{array}{ccc} \lambda_{1}^{2} & 0 & 0\\ 0 & \lambda_{2}^{2} & 0\\ 0 & 0 & \lambda_{3}^{2} \end{array}\right]\;\text{and}\;\dot{\text{C}}=\left[\begin{array}{ccc} 2\lambda_{1}{\dot{\lambda }}_{1} & 0 & 0\\ 0 & 2\lambda_{2}{\dot{\lambda }}_{2} & 0\\ 0 & 0 & 2\lambda_{3}{\dot{\lambda }}_{3} \end{array}\right].$$

Substituting the expressions given in (2.14) into (2.13), and noting that

$$\text{M}⊗\text{M}=\left[\begin{array}{ccc} \cos^{2}\varphi & \cos\varphi \sin\varphi & 0\\ \sin\varphi \cos\varphi & \sin^{2}\varphi & 0\\ 0 & 0 & 0 \end{array}\right],$$

the Cauchy stress components may be established and expressed as

2.15 $$\begin{array}{r} \begin{array}{rl} \sigma_{11} & =-p+2\lambda_{1}^{2}{\left(W_{e}\right)}_{1}+2\lambda_{1}^{2}(\lambda_{2}^{2}+\lambda_{3}^{2}){\left(W_{e}\right)}_{2}+2\lambda_{1}^{2}{\left(W_{e}\right)}_{4}\cos^{2}\varphi +4\lambda_{1}^{4}{\left(W_{e}\right)}_{5}\cos^{2}\varphi \\ & \;+2\lambda_{1}^{2}{\left(W_{v}\right)}_{1}+8\lambda_{1}^{3}{\dot{\lambda }}_{1}{\left(W_{v}\right)}_{2}+8\lambda_{1}^{2}\lambda_{2}\lambda_{3}{\dot{\lambda }}_{2}{\dot{\lambda }}_{3}{\left(W_{v}\right)}_{3}\\ & \;+2\lambda_{1}^{2}{\left(W_{v}\right)}_{4}\cos^{2}\varphi +8\lambda_{1}^{3}{\dot{\lambda }}_{1}{\left(W_{v}\right)}_{5}\cos^{2}\varphi \\ & \;+2\lambda_{1}^{4}{\left(W_{v}\right)}_{6}+8\lambda_{1}^{5}{\dot{\lambda }}_{1}{\left(W_{v}\right)}_{7}+2\lambda_{1}^{6}{\left(W_{v}\right)}_{8}+8\lambda_{1}^{7}{\dot{\lambda }}_{1}{\left(W_{v}\right)}_{9}+2\lambda_{1}^{4}{\left(W_{v}\right)}_{10}\cos^{2}\varphi \\ & \;+8\lambda_{1}^{5}{\dot{\lambda }}_{1}{\left(W_{v}\right)}_{11}\cos^{2}\varphi +2\lambda_{1}^{6}{\left(W_{v}\right)}_{12}\cos^{2}\varphi ,\\ \sigma_{12} & =\lambda_{1}\lambda_{2}{\left(W_{e}\right)}_{4}\sin2\varphi +\lambda_{1}\lambda_{2}(\lambda_{1}^{2}+\lambda_{2}^{2}){\left(W_{e}\right)}_{5}\sin2\varphi \\ & \;+\lambda_{1}\lambda_{2}{\left(W_{v}\right)}_{4}\sin2\varphi +2\lambda_{1}\lambda_{2}(\lambda_{1}{\dot{\lambda }}_{1}+\lambda_{2}{\dot{\lambda }}_{2}){\left(W_{v}\right)}_{5}\sin2\varphi +\lambda_{1}\lambda_{2}^{3}{\left(W_{v}\right)}_{10}\sin2\varphi \\ & \;+2\lambda_{1}^{2}\lambda_{2}^{2}(\lambda_{1}{\dot{\lambda }}_{2}+\lambda_{2}{\dot{\lambda }}_{1}){\left(W_{v}\right)}_{11}\sin2\varphi +\lambda_{1}\lambda_{2}^{5}{\left(W_{v}\right)}_{12}\sin2\varphi ,\\ \sigma_{21} & =\lambda_{1}\lambda_{2}{\left(W_{e}\right)}_{4}\sin2\varphi +\lambda_{1}\lambda_{2}(\lambda_{1}^{2}+\lambda_{2}^{2}){\left(W_{e}\right)}_{5}\sin2\varphi \\ & \;+\lambda_{1}\lambda_{2}{\left(W_{v}\right)}_{4}\sin2\varphi +2\lambda_{1}\lambda_{2}(\lambda_{1}{\dot{\lambda }}_{1}+\lambda_{2}{\dot{\lambda }}_{2}){\left(W_{v}\right)}_{5}\sin2\varphi +\lambda_{1}^{3}\lambda_{2}{\left(W_{v}\right)}_{10}\sin2\varphi \\ & \;+2\lambda_{1}^{2}\lambda_{2}^{2}(\lambda_{1}{\dot{\lambda }}_{2}+\lambda_{2}{\dot{\lambda }}_{1}){\left(W_{v}\right)}_{11}\sin2\varphi +\lambda_{1}^{5}\lambda_{2}{\left(W_{v}\right)}_{12}\sin2\varphi ,\\ \sigma_{22} & =-p+2\lambda_{2}^{2}{\left(W_{e}\right)}_{1}+2\lambda_{2}^{2}(\lambda_{1}^{2}+\lambda_{3}^{2}){\left(W_{e}\right)}_{2}+2\lambda_{2}^{2}{\left(W_{e}\right)}_{4}\sin^{2}\varphi +4\lambda_{2}^{4}{\left(W_{e}\right)}_{5}\sin^{2}\varphi \\ & \;+2\lambda_{2}^{2}{\left(W_{v}\right)}_{1}+8\lambda_{2}^{3}{\dot{\lambda }}_{2}{\left(W_{v}\right)}_{2}+8\lambda_{1}\lambda_{2}^{2}\lambda_{3}{\dot{\lambda }}_{1}{\dot{\lambda }}_{3}{\left(W_{v}\right)}_{3}+2\lambda_{2}^{2}{\left(W_{v}\right)}_{4}\sin^{2}\varphi +8\lambda_{2}^{3}{\dot{\lambda }}_{2}{\left(W_{v}\right)}_{5}\sin^{2}\varphi \\ & \;+2\lambda_{2}^{4}{\left(W_{v}\right)}_{6}+8\lambda_{2}^{5}{\dot{\lambda }}_{2}{\left(W_{v}\right)}_{7}+2\lambda_{2}^{6}{\left(W_{v}\right)}_{8}+8\lambda_{2}^{7}{\dot{\lambda }}_{2}{\left(W_{v}\right)}_{9}+2\lambda_{2}^{4}{\left(W_{v}\right)}_{10}\sin^{2}\varphi \\ & \;+8\lambda_{2}^{5}{\dot{\lambda }}_{2}{\left(W_{v}\right)}_{11}\sin^{2}\varphi +2\lambda_{2}^{6}{\left(W_{v}\right)}_{12}\sin^{2}\varphi \\ \text{and}\;\sigma_{33} & =-p+2\lambda_{3}^{2}{\left(W_{e}\right)}_{1}+2\lambda_{3}^{2}(\lambda_{1}^{2}+\lambda_{2}^{2}){\left(W_{e}\right)}_{2}+2\lambda_{3}^{2}{\left(W_{v}\right)}_{1}+8\lambda_{3}^{3}{\dot{\lambda }}_{3}{\left(W_{v}\right)}_{2}+8\lambda_{1}\lambda_{2}\lambda_{3}^{2}{\dot{\lambda }}_{1}{\dot{\lambda }}_{2}{\left(W_{v}\right)}_{3}\\ & \;+2\lambda_{3}^{4}{\left(W_{v}\right)}_{6}+8\lambda_{3}^{5}{\dot{\lambda }}_{3}{\left(W_{v}\right)}_{7}+2\lambda_{3}^{6}{\left(W_{v}\right)}_{8}+8\lambda_{3}^{7}{\dot{\lambda }}_{3}{\left(W_{v}\right)}_{9}, \end{array}\} \end{array}$$

with *σ*_13_ = *σ*_23_ = 0.

#### 2.5.1. Point of caution

The principle of conservation of angular momentum for a continuum in static equilibrium enforces symmetry upon the Cauchy stress tensor; that is, *σ*_12_ = *σ*_21_. We note that in equation (2.15) this is achieved only if ${\left(W_{v}\right)}_{12}=-{\left(W_{v}\right)}_{10}\text{/}(\lambda_{1}^{2}+\lambda_{2}^{2}).$ Therefore, in order for the Cauchy stress tensor of a transversely isotropic viscoelastic material constructed from *W*_e_ and *W*_v_ functions, defined by the invariants in equations (2.7) and (2.8), to comply with the principle of conservation of angular momentum and hence be symmetrical, the function *W*_v_ must be such that the above relationship between (*W*_v_)_10_ and (*W*_v_)_12_ holds. This point has been overlooked in the literature concerning anisotropic viscoelastic constitutive models developed for application to soft tissues. Models that are derived based on theoretical criteria that do not ascertain the symmetry of the Cauchy stress tensor inevitably describe unrealistic and infeasible stress–deformation relationships and subsequently result in erroneous parameter estimations.

In the light of the interrelationship between (*W*_v_)_10_ and (*W*_v_)_12_, the components of the Cauchy stress tensor given in equation (2.15) may be presented as

2.16 $$\begin{array}{rl} \sigma_{11} & =-p+2\lambda_{1}^{2}{\left(W_{e}\right)}_{1}+2\lambda_{1}^{2}(\lambda_{2}^{2}+\lambda_{3}^{2}){\left(W_{e}\right)}_{2}+2\lambda_{1}^{2}{\left(W_{e}\right)}_{4}\cos^{2}\varphi +4\lambda_{1}^{4}{\left(W_{e}\right)}_{5}\cos^{2}\varphi +2\lambda_{1}^{2}{\left(W_{v}\right)}_{1}\\ & \;+8\lambda_{1}^{3}{\dot{\lambda }}_{1}{\left(W_{v}\right)}_{2}+8\lambda_{1}^{2}\lambda_{2}\lambda_{3}{\dot{\lambda }}_{2}{\dot{\lambda }}_{3}{\left(W_{v}\right)}_{3}+2\lambda_{1}^{2}{\left(W_{v}\right)}_{4}\cos^{2}\varphi +8\lambda_{1}^{3}{\dot{\lambda }}_{1}{\left(W_{v}\right)}_{5}\cos^{2}\varphi +2\lambda_{1}^{4}{\left(W_{v}\right)}_{6}\\ & \;+8\lambda_{1}^{5}{\dot{\lambda }}_{1}{\left(W_{v}\right)}_{7}+2\lambda_{1}^{6}{\left(W_{v}\right)}_{8}+8\lambda_{1}^{7}{\dot{\lambda }}_{1}{\left(W_{v}\right)}_{9}+\frac{2\lambda_{1}^{4}\lambda_{2}^{2}}{\lambda_{1}^{2}+\lambda_{2}^{2}}{\left(W_{v}\right)}_{10}\cos^{2}\varphi +8\lambda_{1}^{5}{\dot{\lambda }}_{1}{\left(W_{v}\right)}_{11}\cos^{2}\varphi ,\\ \sigma_{12} & =\sigma_{21}=\lambda_{1}\lambda_{2}{\left(W_{e}\right)}_{4}\sin2\varphi +\lambda_{1}\lambda_{2}(\lambda_{1}^{2}+\lambda_{2}^{2}){\left(W_{e}\right)}_{5}\sin2\varphi +\lambda_{1}\lambda_{2}{\left(W_{v}\right)}_{4}\sin2\varphi +2\lambda_{1}\lambda_{2}(\lambda_{1}{\dot{\lambda }}_{1}\\ & \;+\lambda_{2}{\dot{\lambda }}_{2}){\left(W_{v}\right)}_{5}\sin2\varphi +\frac{\lambda_{1}^{3}\lambda_{2}^{3}}{\lambda_{1}^{2}+\lambda_{2}^{2}}{\left(W_{v}\right)}_{10}\sin2\varphi +2\lambda_{1}^{2}\lambda_{2}^{2}(\lambda_{1}{\dot{\lambda }}_{2}+\lambda_{2}{\dot{\lambda }}_{1}){\left(W_{v}\right)}_{11}\sin2\varphi ,\\ \sigma_{22} & =-p+2\lambda_{2}^{2}{\left(W_{e}\right)}_{1}+2\lambda_{2}^{2}(\lambda_{1}^{2}+\lambda_{3}^{2}){\left(W_{e}\right)}_{2}+2\lambda_{2}^{2}{\left(W_{e}\right)}_{4}\sin^{2}\varphi +4\lambda_{2}^{4}{\left(W_{e}\right)}_{5}\sin^{2}\varphi \\ & \;+2\lambda_{2}^{2}{\left(W_{v}\right)}_{1}+8\lambda_{2}^{3}{\dot{\lambda }}_{2}{\left(W_{v}\right)}_{2}+8\lambda_{1}\lambda_{2}^{2}\lambda_{3}{\dot{\lambda }}_{1}{\dot{\lambda }}_{3}{\left(W_{v}\right)}_{3}+2\lambda_{2}^{2}{\left(W_{v}\right)}_{4}\sin^{2}\varphi +8\lambda_{2}^{3}{\dot{\lambda }}_{2}{\left(W_{v}\right)}_{5}\sin^{2}\varphi \\ & \;+2\lambda_{2}^{4}{\left(W_{v}\right)}_{6}+8\lambda_{2}^{5}{\dot{\lambda }}_{2}{\left(W_{v}\right)}_{7}+2\lambda_{2}^{6}{\left(W_{v}\right)}_{8}+8\lambda_{2}^{7}{\dot{\lambda }}_{2}{\left(W_{v}\right)}_{9}+\frac{2\lambda_{1}^{2}\lambda_{2}^{4}}{\lambda_{1}^{2}+\lambda_{2}^{2}}{\left(W_{v}\right)}_{10}\sin^{2}\varphi \\ & \;+8\lambda_{2}^{5}{\dot{\lambda }}_{2}{\left(W_{v}\right)}_{11}\sin^{2}\varphi \\ \text{and}\;\sigma_{33} & =-p+2\lambda_{3}^{2}{\left(W_{e}\right)}_{1}+2\lambda_{3}^{2}(\lambda_{1}^{2}+\lambda_{2}^{2}){\left(W_{e}\right)}_{2}+2\lambda_{3}^{2}{\left(W_{v}\right)}_{1}+8\lambda_{3}^{3}{\dot{\lambda }}_{3}{\left(W_{v}\right)}_{2}+8\lambda_{1}\lambda_{2}\lambda_{3}^{2}{\dot{\lambda }}_{1}{\dot{\lambda }}_{2}{\left(W_{v}\right)}_{3}\\ & \;+2\lambda_{3}^{4}{\left(W_{v}\right)}_{6}+8\lambda_{3}^{5}{\dot{\lambda }}_{3}{\left(W_{v}\right)}_{7}+2\lambda_{3}^{6}{\left(W_{v}\right)}_{8}+8\lambda_{3}^{7}{\dot{\lambda }}_{3}{\left(W_{v}\right)}_{9}, \end{array}\}$$

with *σ*_13_ = *σ*_23_ = 0. Additionally, owing to incompressibility, *λ*_3_ = 1/*λ*_1_*λ*_2_; however, for simplicity, we leave *λ*_3_ as it is shown in the expressions (2.16). The expressions in equation (2.16) describe the Cauchy stresses in a general case. We note that, considering only the elastic contribution, these expressions are similar to those presented by Ogden in [18], equations (65)–(68).

### 2.6. Application to biaxial tensile deformation

Biaxial tensile tests characterizing the mechanical behaviour of the AV tissue overwhelmingly use square specimens cut from the central region of the cusp (e.g. [4,5], as shown in figure 2*b*). Subsequently, the specimens have been considered as thin sheet ‘membranes’, and therefore appropriate ensuing assumptions are applied to model the experimental data using mathematical expressions. One such assumption is that, for a thin sheet membrane, the through-thickness (principal) Cauchy stress can be approximated to zero, *σ*_33_ = 0. The expressions in equation (2.16) therefore may be reduced to

2.17 $$\begin{array}{rl} \sigma_{11} & =-p+2\lambda_{1}^{2}{\left(W_{e}\right)}_{1}+2\lambda_{1}^{2}(\lambda_{2}^{2}+\lambda_{3}^{2}){\left(W_{e}\right)}_{2}+2\lambda_{1}^{2}{\left(W_{e}\right)}_{4}\cos^{2}\varphi +4\lambda_{1}^{4}{\left(W_{e}\right)}_{5}\cos^{2}\varphi \\ & \;+2\lambda_{1}^{2}{\left(W_{v}\right)}_{1}+8\lambda_{1}^{3}{\dot{\lambda }}_{1}{\left(W_{v}\right)}_{2}+8\lambda_{1}^{2}\lambda_{2}\lambda_{3}{\dot{\lambda }}_{2}{\dot{\lambda }}_{3}{\left(W_{v}\right)}_{3}+2\lambda_{1}^{2}{\left(W_{v}\right)}_{4}\cos^{2}\varphi +8\lambda_{1}^{3}{\dot{\lambda }}_{1}{\left(W_{v}\right)}_{5}\cos^{2}\varphi \\ & \;+2\lambda_{1}^{4}{\left(W_{v}\right)}_{6}+8\lambda_{1}^{5}{\dot{\lambda }}_{1}{\left(W_{v}\right)}_{7}+2\lambda_{1}^{6}{\left(W_{v}\right)}_{8}+8\lambda_{1}^{7}{\dot{\lambda }}_{1}{\left(W_{v}\right)}_{9}+\frac{2\lambda_{1}^{4}\lambda_{2}^{2}}{\lambda_{1}^{2}+\lambda_{2}^{2}}{\left(W_{v}\right)}_{10}\cos^{2}\varphi \\ & \;+8\lambda_{1}^{5}{\dot{\lambda }}_{1}{\left(W_{v}\right)}_{11}\cos^{2}\varphi ,\\ \sigma_{12} & =\sigma_{21}=\lambda_{1}\lambda_{2}{\left(W_{e}\right)}_{4}\sin2\varphi +\lambda_{1}\lambda_{2}(\lambda_{1}^{2}+\lambda_{2}^{2}){\left(W_{e}\right)}_{5}\sin2\varphi \\ & \;+\lambda_{1}\lambda_{2}{\left(W_{v}\right)}_{4}\sin2\varphi +2\lambda_{1}\lambda_{2}(\lambda_{1}{\dot{\lambda }}_{1}+\lambda_{2}{\dot{\lambda }}_{2}){\left(W_{v}\right)}_{5}\sin2\varphi +\frac{\lambda_{1}^{3}\lambda_{2}^{3}}{\lambda_{1}^{2}+\lambda_{2}^{2}}{\left(W_{v}\right)}_{10}\sin2\varphi \\ & \;+2\lambda_{1}^{2}\lambda_{2}^{2}(\lambda_{1}{\dot{\lambda }}_{2}+\lambda_{2}{\dot{\lambda }}_{1}){\left(W_{v}\right)}_{11}\sin2\varphi \\ \text{and}\;\sigma_{22} & =-p+2\lambda_{2}^{2}{\left(W_{e}\right)}_{1}+2\lambda_{2}^{2}(\lambda_{1}^{2}+\lambda_{3}^{2}){\left(W_{e}\right)}_{2}+2\lambda_{2}^{2}{\left(W_{e}\right)}_{4}\sin^{2}\varphi +4\lambda_{2}^{4}{\left(W_{e}\right)}_{5}\sin^{2}\varphi \\ & \;+2\lambda_{2}^{2}{\left(W_{v}\right)}_{1}+8\lambda_{2}^{3}{\dot{\lambda }}_{2}{\left(W_{v}\right)}_{2}+8\lambda_{1}\lambda_{2}^{2}\lambda_{3}{\dot{\lambda }}_{1}{\dot{\lambda }}_{3}{\left(W_{v}\right)}_{3}+2\lambda_{2}^{2}{\left(W_{v}\right)}_{4}\sin^{2}\varphi +8\lambda_{2}^{3}{\dot{\lambda }}_{2}{\left(W_{v}\right)}_{5}\sin^{2}\varphi \\ & \;+2\lambda_{2}^{4}{\left(W_{v}\right)}_{6}+8\lambda_{2}^{5}{\dot{\lambda }}_{2}{\left(W_{v}\right)}_{7}+2\lambda_{2}^{6}{\left(W_{v}\right)}_{8}+8\lambda_{2}^{7}{\dot{\lambda }}_{2}{\left(W_{v}\right)}_{9}+\frac{2\lambda_{1}^{2}\lambda_{2}^{4}}{\lambda_{1}^{2}+\lambda_{2}^{2}}{\left(W_{v}\right)}_{10}\sin^{2}\varphi \\ & \;+8\lambda_{2}^{5}{\dot{\lambda }}_{2}{\left(W_{v}\right)}_{11}\sin^{2}\varphi . \end{array}\}$$

We note that the hydrostatic pressure *p* can now be determined from *σ*_33_ = 0. Alternatively, following Ogden [18], the expressions in equation (2.16) may be rewritten as

2.18 $$\begin{array}{rl} \sigma_{11}-\sigma_{33} & =2(\lambda_{1}^{2}-\lambda_{3}^{2}){\left(W_{e}\right)}_{1}+2\lambda_{2}^{2}(\lambda_{1}^{2}-\lambda_{3}^{2}){\left(W_{e}\right)}_{2}+2\lambda_{1}^{2}{\left(W_{e}\right)}_{4}\cos^{2}\varphi +4\lambda_{1}^{4}{\left(W_{e}\right)}_{5}\cos^{2}\varphi \\ & \;+2(\lambda_{1}^{2}-\lambda_{3}^{2}){\left(W_{v}\right)}_{1}+8(\lambda_{1}^{3}{\dot{\lambda }}_{1}-\lambda_{3}^{3}{\dot{\lambda }}_{3}){\left(W_{v}\right)}_{2}+8{\dot{\lambda }}_{2}(\lambda_{1}{\dot{\lambda }}_{3}-\lambda_{3}{\dot{\lambda }}_{1}){\left(W_{v}\right)}_{3}\\ & \;+2\lambda_{1}^{2}{\left(W_{v}\right)}_{4}\cos^{2}\varphi +8\lambda_{1}^{3}{\dot{\lambda }}_{1}{\left(W_{v}\right)}_{5}\cos^{2}\varphi +2(\lambda_{1}^{4}-\lambda_{3}^{4}){\left(W_{v}\right)}_{6}+8(\lambda_{1}^{5}{\dot{\lambda }}_{1}-\lambda_{3}^{5}{\dot{\lambda }}_{3}){\left(W_{v}\right)}_{7}\\ & \;+2(\lambda_{1}^{6}-\lambda_{3}^{6}){\left(W_{v}\right)}_{8}+8(\lambda_{1}^{7}{\dot{\lambda }}_{1}-\lambda_{3}^{7}{\dot{\lambda }}_{3}){\left(W_{v}\right)}_{9}+\frac{2\lambda_{1}^{4}\lambda_{2}^{2}}{\lambda_{1}^{2}+\lambda_{2}^{2}}{\left(W_{v}\right)}_{10}\cos^{2}\varphi \\ & \;+8\lambda_{1}^{5}{\dot{\lambda }}_{1}{\left(W_{v}\right)}_{11}\cos^{2}\varphi ,\\ \sigma_{12}=\sigma_{21} & =\lambda_{1}\lambda_{2}{\left(W_{e}\right)}_{4}\sin2\varphi +\lambda_{1}\lambda_{2}(\lambda_{1}^{2}+\lambda_{2}^{2}){\left(W_{e}\right)}_{5}\sin2\varphi +\lambda_{1}\lambda_{2}{\left(W_{v}\right)}_{4}\sin2\varphi \\ & \;+2\lambda_{1}\lambda_{2}(\lambda_{1}{\dot{\lambda }}_{1}+\lambda_{2}{\dot{\lambda }}_{2}){\left(W_{v}\right)}_{5}\sin2\varphi +2\lambda_{1}^{2}\lambda_{2}^{2}(\lambda_{1}{\dot{\lambda }}_{2}+\lambda_{2}{\dot{\lambda }}_{1}){\left(W_{v}\right)}_{11}\sin2\varphi \\ \text{and}\;\sigma_{22}-\sigma_{33} & =2(\lambda_{2}^{2}-\lambda_{3}^{2}){\left(W_{e}\right)}_{1}+2\lambda_{1}^{2}(\lambda_{2}^{2}-\lambda_{3}^{2}){\left(W_{e}\right)}_{2}+2\lambda_{2}^{2}{\left(W_{e}\right)}_{4}\sin^{2}\varphi +4\lambda_{2}^{4}{\left(W_{e}\right)}_{5}\sin^{2}\varphi \\ & \;+2(\lambda_{2}^{2}-\lambda_{3}^{2}){\left(W_{v}\right)}_{1}+8(\lambda_{2}^{3}{\dot{\lambda }}_{2}-\lambda_{3}^{3}{\dot{\lambda }}_{3}){\left(W_{v}\right)}_{2}+8{\dot{\lambda }}_{1}(\lambda_{2}{\dot{\lambda }}_{3}-\lambda_{3}{\dot{\lambda }}_{2}){\left(W_{v}\right)}_{3}\\ & \;+2\lambda_{2}^{2}{\left(W_{v}\right)}_{4}\sin^{2}\varphi +8\lambda_{2}^{3}{\dot{\lambda }}_{2}{\left(W_{v}\right)}_{5}\sin^{2}\varphi +2(\lambda_{2}^{4}-\lambda_{3}^{4}){\left(W_{v}\right)}_{6}+8(\lambda_{2}^{5}{\dot{\lambda }}_{2}-\lambda_{3}^{5}{\dot{\lambda }}_{3}){\left(W_{v}\right)}_{7}\\ & \;+2(\lambda_{2}^{6}-\lambda_{3}^{6}){\left(W_{v}\right)}_{8}+8(\lambda_{2}^{7}{\dot{\lambda }}_{2}-\lambda_{3}^{7}{\dot{\lambda }}_{3}){\left(W_{v}\right)}_{9}+\frac{2\lambda_{1}^{2}\lambda_{2}^{4}}{\lambda_{1}^{2}+\lambda_{2}^{2}}{\left(W_{v}\right)}_{10}\sin^{2}\varphi \\ & \;+8\lambda_{2}^{5}{\dot{\lambda }}_{2}{\left(W_{v}\right)}_{11}\sin^{2}\varphi , \end{array}\}$$

where, again, for a thin sheet membrane, the through-thickness (principal) Cauchy stress can be approximated to zero *σ*_33_ = 0.

Notwithstanding the viscous terms in the expressions in equations (2.17) and (2.18), (i.e. terms that include (*W*_v_)*_i_*), equations (2.18) render three independent components of deformation and stress, while containing four (*W*_e_)*_i_* terms. Therefore, four equations are required to characterize the *W*_e_ function, and thereby the properties of the AV tissue, while biaxial tensile tests at best could provide information regarding three independent deformation–stress sets. This problem has been discussed and analysed at length by Holzapfel & Ogden [18,19]. Equations (2.17) and (2.18) suggest that this problem is further exacerbated by inclusion of viscous terms. From this perspective, therefore, biaxial tests do not have much advantage over other loading protocols that may render lower ranks of datasets than the number of unknowns [20], particularly in characterizing the anisotropic viscoelastic behaviour of soft tissues such as the AV.

It must be further noted that experimental systems that could facilitate independent control of in-plane shear have not yet been introduced and employed in performing the tensile deformation tests on AV tissue specimens, as reflected in recent reviews of the state of the art [7,29,30]. Moreover, in the light of equation (2.18), if the preferred fibre direction is along one of the coordinate axes, i.e. *φ* = 0 or *φ* = *π*/2, then *σ*_12_ = *σ*_21_ = 0. This is often the case in square and/or rectangular specimens used in biaxial and uniaxial deformation tests of the AV, prepared from the valve cusps as shown in figure 2. Therefore, expressions in equation (2.18) may be reduced to

2.19 $$\begin{array}{rl} \sigma_{11} & =2(\lambda_{1}^{2}-\lambda_{3}^{2}){\left(W_{e}\right)}_{1}+2\lambda_{2}^{2}(\lambda_{1}^{2}-\lambda_{3}^{2}){\left(W_{e}\right)}_{2}+2\lambda_{1}^{2}{\left(W_{e}\right)}_{4}\cos^{2}\varphi +4\lambda_{1}^{4}{\left(W_{e}\right)}_{5}\cos^{2}\varphi \\ & \;+2(\lambda_{1}^{2}-\lambda_{3}^{2}){\left(W_{v}\right)}_{1}+8(\lambda_{1}^{3}{\dot{\lambda }}_{1}-\lambda_{3}^{3}{\dot{\lambda }}_{3}){\left(W_{v}\right)}_{2}+8{\dot{\lambda }}_{2}(\lambda_{1}{\dot{\lambda }}_{3}-\lambda_{3}{\dot{\lambda }}_{1}){\left(W_{v}\right)}_{3}\\ & \;+2\lambda_{1}^{2}{\left(W_{v}\right)}_{4}\cos^{2}\varphi +8\lambda_{1}^{3}{\dot{\lambda }}_{1}{\left(W_{v}\right)}_{5}\cos^{2}\varphi +2(\lambda_{1}^{4}-\lambda_{3}^{4}){\left(W_{v}\right)}_{6}+8(\lambda_{1}^{5}{\dot{\lambda }}_{1}-\lambda_{3}^{5}{\dot{\lambda }}_{3}){\left(W_{v}\right)}_{7}\\ & \;+2(\lambda_{1}^{6}-\lambda_{3}^{6}){\left(W_{v}\right)}_{8}+8(\lambda_{1}^{7}{\dot{\lambda }}_{1}-\lambda_{3}^{7}{\dot{\lambda }}_{3}){\left(W_{v}\right)}_{9}+\frac{2\lambda_{1}^{4}\lambda_{2}^{2}}{\lambda_{1}^{2}+\lambda_{2}^{2}}{\left(W_{v}\right)}_{10}\cos^{2}\varphi +8\lambda_{1}^{5}{\dot{\lambda }}_{1}{\left(W_{v}\right)}_{11}\cos^{2}\varphi \\ \text{and}\;\sigma_{22} & =2(\lambda_{2}^{2}-\lambda_{3}^{2}){\left(W_{e}\right)}_{1}+2\lambda_{1}^{2}(\lambda_{2}^{2}-\lambda_{3}^{2}){\left(W_{e}\right)}_{2}+2\lambda_{2}^{2}{\left(W_{e}\right)}_{4}\sin^{2}\varphi +4\lambda_{2}^{4}{\left(W_{e}\right)}_{5}\sin^{2}\varphi \\ & \;+2(\lambda_{2}^{2}-\lambda_{3}^{2}){\left(W_{v}\right)}_{1}+8(\lambda_{2}^{3}{\dot{\lambda }}_{2}-\lambda_{3}^{3}{\dot{\lambda }}_{3}){\left(W_{v}\right)}_{2}+8{\dot{\lambda }}_{1}(\lambda_{2}{\dot{\lambda }}_{3}-\lambda_{3}{\dot{\lambda }}_{2}){\left(W_{v}\right)}_{3}\\ & \;+2\lambda_{2}^{2}{\left(W_{v}\right)}_{4}\sin^{2}\varphi +8\lambda_{2}^{3}{\dot{\lambda }}_{2}{\left(W_{v}\right)}_{5}\sin^{2}\varphi +2(\lambda_{2}^{4}-\lambda_{3}^{4}){\left(W_{v}\right)}_{6}+8(\lambda_{2}^{5}{\dot{\lambda }}_{2}-\lambda_{3}^{5}{\dot{\lambda }}_{3}){\left(W_{v}\right)}_{7}\\ & \;+2(\lambda_{2}^{6}-\lambda_{3}^{6}){\left(W_{v}\right)}_{8}+8(\lambda_{2}^{7}{\dot{\lambda }}_{2}-\lambda_{3}^{7}{\dot{\lambda }}_{3}){\left(W_{v}\right)}_{9}+\frac{2\lambda_{1}^{2}\lambda_{2}^{4}}{\lambda_{1}^{2}+\lambda_{2}^{2}}{\left(W_{v}\right)}_{10}\sin^{2}\varphi +8\lambda_{2}^{5}{\dot{\lambda }}_{2}{\left(W_{v}\right)}_{11}\sin^{2}\varphi . \end{array}\}$$

### 2.7. Application to uniaxial tensile deformation

In uniaxial tests, rectangular strips are cut from the central region of the valve cusp along the preferred fibre direction (circumferential), and the transverse direction (radial), as shown schematically in figure 2*b*. For a circumferential strip under uniaxial tensile deformation along that direction, *σ*_22_ = 0 and *φ* = 0°, as shown in figure 2*c*. Therefore, the principal Cauchy stress in circumferential direction is established from equation (2.19) as

2.20 $$\begin{array}{rl} \sigma_{11}=\sigma_{\mathrm{circumferential}} & =2(\lambda_{1}^{2}-\lambda_{3}^{2}){\left(W_{e}\right)}_{1}+2\lambda_{2}^{2}(\lambda_{1}^{2}-\lambda_{3}^{2}){\left(W_{e}\right)}_{2}+2\lambda_{1}^{2}{\left(W_{e}\right)}_{4}+4\lambda_{1}^{4}{\left(W_{e}\right)}_{5}\\ & \;+2(\lambda_{1}^{2}-\lambda_{3}^{2}){\left(W_{v}\right)}_{1}+8(\lambda_{1}^{3}{\dot{\lambda }}_{1}-\lambda_{3}^{3}{\dot{\lambda }}_{3}){\left(W_{v}\right)}_{2}+8{\dot{\lambda }}_{2}(\lambda_{1}{\dot{\lambda }}_{3}-\lambda_{3}{\dot{\lambda }}_{1}){\left(W_{v}\right)}_{3}\\ & \;+2\lambda_{1}^{2}{\left(W_{v}\right)}_{4}+8\lambda_{1}^{3}{\dot{\lambda }}_{1}{\left(W_{v}\right)}_{5}+2(\lambda_{1}^{4}-\lambda_{3}^{4}){\left(W_{v}\right)}_{6}+8(\lambda_{1}^{5}{\dot{\lambda }}_{1}-\lambda_{3}^{5}{\dot{\lambda }}_{3}){\left(W_{v}\right)}_{7}\\ & \;+2(\lambda_{1}^{6}-\lambda_{3}^{6}){\left(W_{v}\right)}_{8}+8(\lambda_{1}^{7}{\dot{\lambda }}_{1}-\lambda_{3}^{7}{\dot{\lambda }}_{3}){\left(W_{v}\right)}_{9}+\frac{2\lambda_{1}^{4}\lambda_{2}^{2}}{\lambda_{1}^{2}+\lambda_{2}^{2}}{\left(W_{v}\right)}_{10}+8\lambda_{1}^{5}{\dot{\lambda }}_{1}{\left(W_{v}\right)}_{11}, \end{array}$$

where *λ*_1_·*λ*_2_·*λ*_3_ = 1 and *λ*_2_ = *λ*_3_. Substituting these into the above, equation (2.20) may be rewritten as

2.21 $$\begin{array}{rl} \sigma_{11}=\sigma_{\mathrm{circumferential}} & =2(\lambda_{1}^{2}-\lambda_{1}^{-1}){\left(W_{e}\right)}_{1}+2(\lambda_{1}-\lambda_{1}^{-2}){\left(W_{e}\right)}_{2}+2\lambda_{1}^{2}{\left(W_{e}\right)}_{4}+4\lambda_{1}^{4}{\left(W_{e}\right)}_{5}\\ & \;+2(\lambda_{1}^{2}-\lambda_{1}^{-1}){\left(W_{v}\right)}_{1}+\text{4}{\dot{\lambda }}_{1}(2\lambda_{1}^{3}+\lambda_{1}^{-3}){\left(W_{v}\right)}_{2}+6\lambda_{1}^{-2}{\dot{\lambda }}_{1}^{2}{\left(W_{v}\right)}_{3}+2\lambda_{1}^{2}{\left(W_{v}\right)}_{4}\\ & \;+8\lambda_{1}^{3}{\dot{\lambda }}_{1}{\left(W_{v}\right)}_{5}+2(\lambda_{1}^{4}-\lambda_{1}^{-2}){\left(W_{v}\right)}_{6}+\text{4}{\dot{\lambda }}_{1}(2\lambda_{1}^{5}+\lambda_{1}^{-4}){\left(W_{v}\right)}_{7}+2(\lambda_{1}^{6}-\lambda_{1}^{-3}){\left(W_{v}\right)}_{8}\\ & \;+4{\dot{\lambda }}_{1}(2\lambda_{1}^{7}+\lambda_{1}^{-5}){\left(W_{v}\right)}_{9}+\frac{2\lambda_{1}^{3}}{\lambda_{1}^{2}+\lambda_{1}^{-1}}{\left(W_{v}\right)}_{10}+8\lambda_{1}^{5}{\dot{\lambda }}_{1}{\left(W_{v}\right)}_{11}. \end{array}$$

Similarly, for a radial strip under uniaxial tensile deformation along that direction, *σ*_11_ = 0, *φ* = 90° as shown in figure 2*c*, and *λ*_1_ = *λ*_3_. Furthermore, because the fibres are aligned in the circumferential direction, we expect negligible contribution from (*W*_e_)_4_ and (*W*_e_)_5_ when the strip is stretched in the radial direction, as the fibres do not support compression [18], and the same may be assumed for the contribution of (*W*_v_)_4_, (*W*_v_)_5_, (*W*_v_)_10_ and (*W*_v_)_11_. The principal Cauchy stress in the radial direction is therefore established from equation (2.19) as

2.22 $$\begin{array}{rl} \sigma_{22}=\sigma_{\mathrm{radial}} & =2(\lambda_{2}^{2}-\lambda_{2}^{-1}){\left(W_{e}\right)}_{1}+2(\lambda_{2}-\lambda_{2}^{-2}){\left(W_{e}\right)}_{2}+2(\lambda_{2}^{2}-\lambda_{2}^{-1}){\left(W_{v}\right)}_{1}\\ & \;+4{\dot{\lambda }}_{2}(2\lambda_{2}^{3}+\lambda_{2}^{-3}){\left(W_{v}\right)}_{2}+6\lambda_{2}^{-2}{\dot{\lambda }}_{2}^{2}{\left(W_{v}\right)}_{3}+2(\lambda_{2}^{4}-\lambda_{2}^{-2}){\left(W_{v}\right)}_{6}\\ & \;+4{\dot{\lambda }}_{2}(2\lambda_{2}^{5}+\lambda_{2}^{-4}){\left(W_{v}\right)}_{7}+2(\lambda_{2}^{6}-\lambda_{2}^{-3}){\left(W_{v}\right)}_{8}+4{\dot{\lambda }}_{2}(2\lambda_{2}^{7}+\lambda_{2}^{-5}){\left(W_{v}\right)}_{9}. \end{array}$$

Equations (2.21) and (2.22) express the principal Cauchy stress components in the circumferential and radial directions, respectively, under uniaxial tensile deformation. However, we note that uniaxial tensile tests provide two independent stress–deformation datasets, whereas equations (2.21) and (2.22) include 15 unknowns, four (*W*_e_)*_i_*s and 11 (*W*_v_)*_i_*s. Therefore, reasonable assumptions and appropriate forms of energy functions shall be considered for specialization of equations (2.21) and (2.22), to enable formulation of admissible models that can suitably describe the stress–deformation data. Such considerations include *a priori* assumptions regarding the appropriate number of invariants in energy functions, as well as certain physical and mathematical conditions which ensure model validity and material stability. In the following, we shall invoke these considerations and introduce the energy functions in mathematical form.

## 3. Model formulation

### 3.1. *W*_e_ and *W*_v_ functions

As equations (2.19), (2.21) and (2.22) indicate, biaxial tensile tests in which only two strain components are varied independently, and uniaxial tensile tests in transverse directions, inherently do not provide enough independent datasets for a complete characterization of energy functions *W* in transversely isotropic viscoelastic tissues. This is mathematically inferred, as the number of constitutive functions (*W*_e_)*_i_* and (*W*_v_)*_i_* supersedes the number of existing relationships between stress and deformation. In such cases, it is admissible to assume *a priori* that *W*_e_ and *W*_v_ are a function of only certain invariants, i.e. some of the invariants are considered absent from the general form of the energy functions [19,22,31,32]. Therefore, one may be faced with the task of choosing an appropriate form of *W*_e_ and *W*_v_.

Standardized theoretical frameworks that facilitate axiomatic choices of elastic and/or viscous energy functions have not been articulated in the literature concerning soft tissues, if, indeed they are possible to develop. For elastic energy functions, Ogden [18] advocates three baseline factors that provide a sound reference for a valid starting point. First, *W*_e_ must be chosen, so that the ensuing stress–deformation relationships are consistent with the experimentally observed behaviour of the subject tissue. For example, most collagenous soft tissues exhibit an initial ‘soft’ stress–deformation phase, followed by a stiffening phase at higher deformations. An appropriate *W*_e_ should therefore accommodate this nonlinear stress–deformation behaviour. Second, *W*_e_ must reflect the relevant material symmetry of the subject tissue. For example, if a tissue is transversely isotropic, an appropriate *W*_e_ function for that tissue must reflect this characteristic material symmetry. Third, *W*_e_ must satisfy the condition of ellipticity and convexity, in order to furnish well-posed boundary-value problems and material stability. For the viscous energy function, thermodynamic requirements enforce *W*_v_ to be continuous, positive and convex with respect to $\dot{\text{C}}$ [22]. In addition, the value of *W*_v_ must be zero when $\dot{\text{C}}$ is equal to zero [22].

Taking the above considerations into account, a widely acceptable elastic energy function *W*_e_ for incompressible transversely isotropic tissues has been devised to depend only on invariants *I*_1_ and *I*_4_, of the following form [19,21,31]:

3.1 $$W_{e}=W_{e}^{\mathrm{iso}}(I_{1})+W_{e}^{\mathrm{fibres}}(I_{4}),$$

where $W_{e}^{\mathrm{iso}}$ and $W_{e}^{\mathrm{fibres}}$ represent the influence of the isotropic matrix and the mean preferred fibre direction, respectively, on the overall elastic behaviour of the AV. For the purpose of our model, we employ an exponential-type elastic energy function for the isotropic matrix $W_{e}^{iso}$ (as advocated in [15]), and a ‘Holzapfel-type’ elastic energy function for the contribution of the embedded fibre family $W_{e}^{\mathrm{fibres}}$ [19,21,31]:

3.2 $$W_{e}=\frac{1}{2}\alpha (\exp[\beta (I_{1}-3)]-1)+\frac{k_{1}}{2k_{2}}(\exp[k_{2}{\left(I_{4}-1\right)}^{2}]-1),$$

where *α* and *k*_1_ are positive stress-like material parameters, and *β* and *k*_2_ are positive dimensionless parameters. We note that there are now only two invariants incorporated in the *W*_e_ function, which, given the fact that biaxial and uniaxial tensile tests in transverse directions provide two independent stress–deformation equations, in principle should enable one to characterize the elastic behaviour of the valve if the elastic response of the tissue specimens is established from the experiments.

For devising an appropriate viscous energy function, we note that the viscous effects of the bulk AV tissue may stem from the gel-like viscous GAG matrix as outlined in §2.2, in addition to the dissipative kinematics of the fibre–matrix and the fibre–fibre sliding and interaction [6]. Therefore, we consider the overall viscous energy function *W*_v_ of the valve as the sum of the contribution of the valve's viscous matrix $W_{v}^{\mathrm{matrix}}$ and the dissipative kinetics of the fibres $W_{v}^{\mathrm{fibres}}.$ Following Pioletti *et al*. [22], we choose $W_{v}^{\mathrm{matrix}}$ to depend on the invariants *I*_1_ and *J*_2_, and assume $W_{v}^{\mathrm{fibres}}$ to depend on the invariants *I*_1_ and *J*_5_, for the viscous energy function *W*_v_ to have the following form:

3.3 $$\begin{array}{rl} W_{v} & =W_{v}^{\mathrm{matrix}}(I_{1},J_{2})+W_{v}^{\mathrm{fibres}}(I_{1},J_{5})\\ & =\frac{\eta_{1}}{4}J_{2}(I_{1}-3)+\frac{\eta_{2}}{4}J_{5}(I_{1}-3)\\ & =\frac{1}{4}(I_{1}-3)(\eta_{1}J_{2}+\eta_{2}J_{5}), \end{array}$$

where *η*_1_ and *η*_2_ are viscosity-like parameters reflecting the dissipative effects of the viscous matrix and the fibre kinematics, respectively, and are positive. We note that according to the definition of *J*_2_ and *J*_5_ given in equation (2.8), *W*_v_ is a quadratic function of $\dot{\text{C}}$ (i.e. $W_{v}=f({\dot{\text{C}}}^{2})$), and therefore is convex in $\dot{\text{C}}.$ Moreover, it may be observed that *W*_v_ = 0 when $\dot{\text{C}}=\text{0}.$ Equation (3.3) introduces two additional invariants (*J*_2_ and *J*_5_) to the stress–deformation equations. However, the only constitutive component of *W*_v_ in equation (3.3) that appears in the stress–deformation equation in the radial direction (equation (2.22)) is (*W*_v_)_2_. Therefore, theoretically, (*W*_v_)_2_ may be characterized using an additional set of stress–deformation data obtained from tensile tests performed under a different strain rate compared with that of the elastic response, in the radial direction. Then, the stress–deformation equation in the circumferential direction (equation (2.21)) facilitates the characterization of (*W*_v_)_5_ using a set of stress–deformation data obtained from tensile tests performed in the same direction but under a different strain rate compared with that of the elastic response. Therefore, from a theoretical point of view, stress–deformation curves obtained from AV specimens under various deformation rates in transverse directions should, in principle, allow characterization of the viscous energy function *W*_v_.

### 3.2. Transversely isotropic viscoelastic model

Equations (3.2) and (3.3) may now be inserted into equation (2.19) to develop a model to describe the stress–deformation behaviour of the AV under biaxial tension or similarly into equations (2.21) and (2.22) for a uniaxial model describing the deformation in transverse directions. For the purpose of this study, we have performed uniaxial tensile tests on AV specimens. We shall therefore employ equations (2.21) and (2.22) to develop a transversely isotropic viscoelastic model applicable to the uniaxial data:

3.4 $$\begin{array}{rl} \sigma_{11}=\sigma_{\mathrm{circumferential}} & =\alpha \beta (\lambda_{1}^{2}-\lambda_{1}^{-1})(\text{exp}[\beta (\lambda_{1}^{2}+2\lambda_{1}^{-1}-3)])+2k_{1}\lambda_{1}^{2}(\lambda_{1}^{2}-1)\text{exp}[k_{2}{\left(\lambda_{1}^{2}-1\right)}^{2}]\\ & \;+{\dot{\lambda }}_{1}(\lambda_{1}^{2}+2\lambda_{1}^{-1}-3)(\eta_{1}[2\lambda_{1}^{3}+\lambda_{1}^{-3}]+2\eta_{2}\lambda_{1}^{3})\\ \text{and}\;\sigma_{22}=\sigma_{\mathrm{radial}} & =\alpha \beta (\lambda_{2}^{2}-\lambda_{2}^{-1})(\text{exp[ }\beta (\lambda_{2}^{2}+2\lambda_{2}^{-1}-3)])+\eta_{1}{\dot{\lambda }}_{2}(2\lambda_{2}^{3}+\lambda_{2}^{-3})(\lambda_{2}^{2}+2\lambda_{2}^{-1}-3). \end{array}\}$$

The expressions in equation (3.4) represent the final form of our transversely isotropic viscoelastic model, describing the stress–deformation behaviour of the AV leaflet, using uniaxial tension in transverse directions.

## 4. Tensile deformation tests

For the purpose of this study, we used experimental stress–deformation data of AV specimens subjected to uniaxial tensile tests to failure in both circumferential and radial directions. The data were obtained under four stretch rates $\dot{\lambda }$ of 0.001, 0.01, 0.1 and 0.5 s^−1^. Porcine hearts (*n* = 10 in total) were obtained from mature animals, ranging from 18 to 24 months, within 2 h of slaughter from a local abattoir. The three AV leaflets were dissected from the aortic root and maintained in Dulbecco's modified Eagle's medium (DMEM, Sigma, Poole, UK) at room temperature (25°C). From each leaflet, a 5 mm wide circumferential or radial strip was excised from the central (belly) region (figure 2*b*).

The tensile tests to failure were performed using two material testing machines, a Bionix 100 (MTS, Cirencester, UK) for tests under $\dot{\lambda }=0.001\;s^{-1},$ $\dot{\lambda }=0.01\;s^{-1}$ and $\dot{\lambda }=0.1\;s^{-1},$ and a Bose Electroforce 3200 (Minnesota, USA) for tests above $\dot{\lambda }=0.1\;s^{-1}.$ The initial distance between the grips was set at 10 mm for all test protocols, after which a tare load of 0.01 N was applied to the specimens, to establish a consistent zero position. The adjusted distance between the grips was then used as the initial sample length. For each tensile test, three specimens were used. No preconditioning was applied to the specimens prior to the start of the tests.

**Figure 3.**
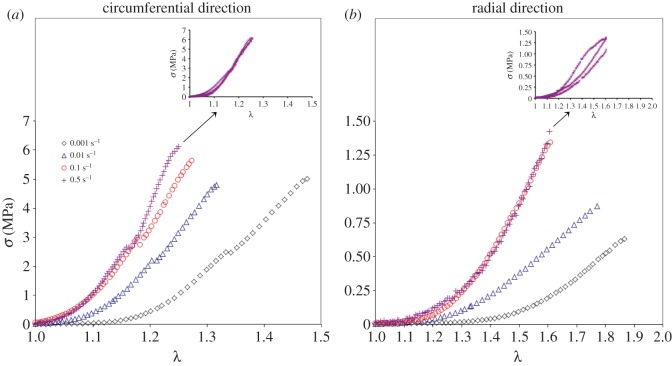
The representative *σ* − *λ* curves for (*a*) circumferentially and (*b*) radially loaded samples subjected to increasing stretch rates $\dot{\lambda }.$ The small panels at the top show the curves obtained from the three tested specimens at $\dot{\lambda }=0.5\;s^{-1},$ as an indicator of the repeatability of the obtained data.

The stress–deformation curves of the AV specimens obtained at $\dot{\lambda }=0.001\;s^{-1},$ $\dot{\lambda }=0.01\;s^{-1}$ and $\dot{\lambda }=0.1\;s^{-1}$ were reported in a previous study [6], and are reproduced here in conjunction with the new data collected specifically for this study, at a stretch rate of $\dot{\lambda }=0.5\;s^{-1}.$ It must be noted that the experimental results obtained from the tensile tests provide data in terms of *λ* and the nominal ‘engineering’ stress **P**. To enable the application of the experimental data to the developed model in equation (3.3), one must first convert the engineering stress **P** to Cauchy stress **σ** via **σ** = **P***λ* [33]. The resulting *σ* − *λ* curves under the corresponding stretch rates are shown in figure 3, for representative circumferentially and radially loaded samples. The data highlight increasing sample stiffness with increasing $\dot{\lambda }.$ However, the strain rate-associated stiffening appears to be rate-limited, especially in the radial direction, suggesting the data are approaching a threshold $\dot{\lambda }$ whereby increasing the deformation rate would not significantly alter the deformation curves.

## 5. Procedure for model application and parameter estimation

### 5.1. Rate dependency of the parameters

**Figure 4.**
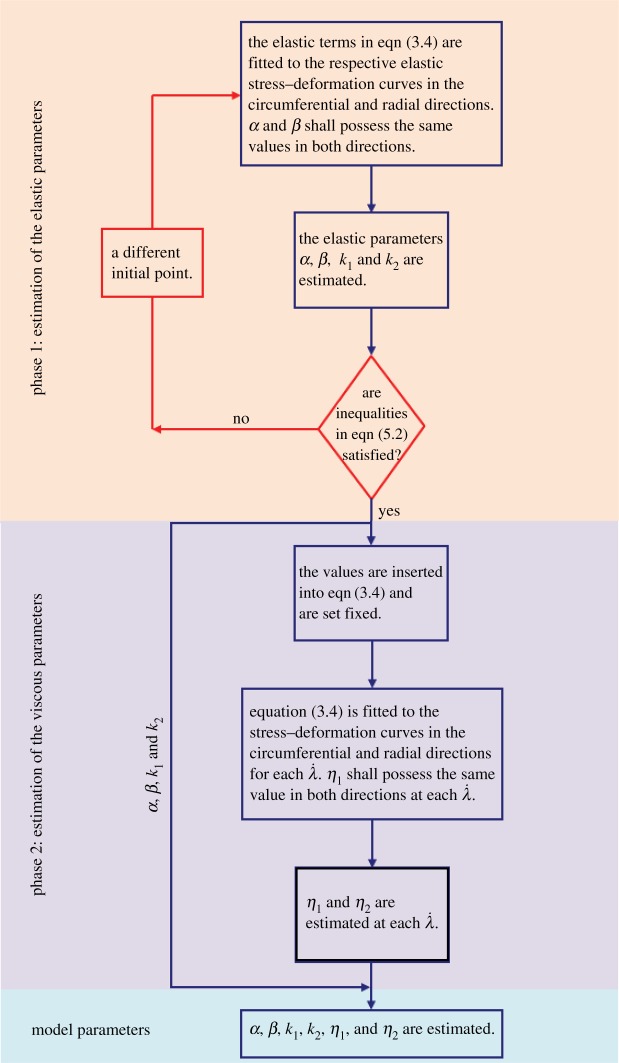
Procedure for the estimation of the model parameters in equation (3.4).

Equation (2.3) associates the overall stress in a viscoelastic continuum to the superposition of the elastic and the viscous contributions of its constituents. The premise of elasticity requires the elastic response of the continuum to be independent of the deformation rate. The viscous effects, by contrast, are dependent on the rate. Therefore, when the model is fitted to the stress–deformation curves obtained at various deformation rates, the parameters related to the elastic behaviour are to remain unchanged, whereas the viscous-related parameters reflect the rate dependency and alter at each rate. To this end, it is important to experimentally establish the elastic response of the tissue, i.e. the elastic stress–deformation curve, from which the associated elastic parameters of the model may be derived. Those parameters are then set to remain unchanged, while fitting the whole model to the stress–deformation curves obtained at different rates, to characterize the viscous-related parameters. The flowchart in figure 4 illustrates this procedure.

### 5.2. Elastic response

It is perhaps impractical to characterize and obtain a ‘pure’ elastic response from tissue samples that are inherently viscoelastic. The stress–strain curves of viscoelastic tissues often exhibit a marked rate dependency, particularly in the case of AV [6]. Pioletti & Rakotomanana [34] postulate that in these circumstances the choice of elastic curve is a matter of definition and identify the curves obtained at lower rates as the elastic response. We qualify this definition further by countenancing the role of characteristic time. Stress–relaxation tests enable the quantification of the characteristic times *τ*, fast and slow, whereby 99% of the relaxation fades within a time *t* = 5*τ* [9]. Therefore, if the deformation rate of the tensile test of tissue specimens is chosen sufficiently low to allow enough time for the viscous processes to take effect and fade, the ensuing stress–deformation curve may be deemed intractable to further reductive viscous effects. Such a curve may therefore provide a baseline that, with a degree of tolerance, may be referred to as the elastic curve.

To apply this definition to our AV samples, we take the average slow relaxation time as reported previously [9] to be *τ*_circum_ = 81.14 s and *τ*_rad_ = 32.11 s, in the circumferential and radial directions respectively. We note that by allowing enough time for the slow relaxation to take effect, the fast relaxation process has taken effect and completed *a priori*. Given that the maximum failure elongation of the specimens is reported to be *λ*_circum_ = 1.46 and *λ*_rad_ = 1.89 [6], a sufficiently low elongation rate to allow for the slow relaxation may be approximated as ${\dot{\lambda }}_{\mathrm{circum}}=0.0011\;s^{-1}$ and ${\dot{\lambda }}_{\mathrm{rad}}=0.0055\;s^{-1},$ assuming a linear relationship between elongation and time. Therefore, an elongation rate of $\dot{\lambda }=0.001\;s^{-1}$ would ensure achieving a stress–deformation curve intractable to additional reductive viscous effects, which we consider as the elastic curve.

### 5.3. Convexity

The common approach to characterize the model parameters *α*, *β*, *k*_1_, *k*_2_, *η*_1_ and *η*_2_ is to fit the model in equation (3.4) to the experimental data obtained from the tensile deformation tests described above. In the process of fitting, however, due care must be observed to ensure that the achieved parameter values do not result in undesirable material instabilities or implausible physical behaviours. Therefore, appropriate mechanically and mathematically motivated constraints need to be derived and applied to restrict the solutions to a meaningful domain in the ‘parameter space’. These constraints are often expressed in the form of mathematical inequalities, imposed on the constitutive parameters during the process of fitting. Following Holzapfel & Ogden, we employ the condition of strict local convexity in order to obtain the relevant inequalities [18,21].

From a mathematical point of view, the condition of strict local convexity requires that the matrix containing the second derivatives of the energy function *W* with respect to the Green–Lagrange strain tensor (**E**), or alternatively with respect to the principal stretches (*λ*), to be positive definite [18,21]. This matrix, also known as the Hessian of *W*, in terms of *λ* is presented by

5.1 $$\text{H}=\left[\begin{array}{cc} \frac{\partial^{2}W}{\partial \lambda_{1}^{2}} & \frac{\partial^{2}W}{\partial \lambda_{1}\partial \lambda_{2}}\\ \frac{\partial^{2}W}{\partial \lambda_{2}\partial \lambda_{1}} & \frac{\partial^{2}W}{\partial \lambda_{2}^{2}} \end{array}\right].$$

We note that **H** in equation (5.1) represents the Hessian matrix and *W* represents the total energy function, i.e. *W*_e_ + *W*_v_. In view of equations (3.2) and (3.3),

$$\frac{\partial^{2}W}{\partial \lambda_{1}\partial \lambda_{2}}=\frac{\partial^{2}W}{\partial \lambda_{2}\partial \lambda_{1}}=\frac{1}{2}\alpha \beta^{2}(\exp[\beta (I_{1}-3)])\frac{\partial I_{1}}{\partial \lambda_{1}}\cdot \frac{\partial I_{1}}{\partial \lambda_{2}}+2k_{1}k_{2}{\left(I_{4}-1\right)}^{2}\exp[k_{2}{\left(I_{4}-1\right)}^{2}]\frac{\partial I_{4}}{\partial \lambda_{1}}\cdot \frac{\partial I_{4}}{\partial \lambda_{2}},$$

which implies that *H* is a symmetric matrix. Therefore, in order for **H** to be positive definite, it is necessary that $\partial^{2}W\text{/}\partial \lambda_{1}^{2},$ $\partial^{2}W\text{/}\partial \lambda_{2}^{2},$ det (**H**) and the eigenvalues of **H** are all positive. Thus

5.2 $$\begin{array}{rl} \frac{\partial^{2}W}{\partial \lambda_{1}^{2}}>0 & \Rightarrow \frac{1}{2}\alpha \beta^{2}(\exp[\beta (I_{1}-3)])\frac{\partial^{2}I_{1}}{\partial \lambda_{1}^{2}}+2k_{1}k_{2}{\left(I_{4}-1\right)}^{2}\exp[k_{2}{\left(I_{4}-1\right)}^{2}]\frac{\partial^{2}I_{4}}{\partial \lambda_{1}^{2}}>0,\\ \frac{\partial^{2}W}{\partial \lambda_{2}^{2}}>0 & \Rightarrow \frac{1}{2}\alpha \beta^{2}(\exp[\beta (I_{1}-3)])\frac{\partial^{2}I_{1}}{\partial \lambda_{2}^{2}}+2k_{1}k_{2}{\left(I_{4}-1\right)}^{2}\exp[k_{2}{\left(I_{4}-1\right)}^{2}]\frac{\partial^{2}I_{4}}{\partial \lambda_{2}^{2}}>0,\\ \det(\text{H})>0 & \Rightarrow \alpha \beta^{2}k_{1}k_{2}{\left(I_{4}-1\right)}^{2}(\exp[\beta (I_{1}-3)])\exp[k_{2}{\left(I_{4}-1\right)}^{2}]\\ & \;\times \left(\frac{\partial^{2}I_{1}}{\partial \lambda_{1}^{2}}\cdot \frac{\partial^{2}I_{4}}{\partial \lambda_{2}^{2}}+\frac{\partial^{2}I_{1}}{\partial \lambda_{2}^{2}}\cdot \frac{\partial^{2}I_{4}}{\partial \lambda_{1}^{2}}\right)>0\\ \text{and}\;\text{eigenvalues}(\text{H})>0 & \Rightarrow \text{both roots of the ‘characteristic equation’ must be positive.} \end{array}\}$$

These inequalities indicate that the material parameters cannot be chosen arbitrarily, and ensure the strict local convexity of *W*. In the light of equation (2.7) and the constraint that *φ* can assume either 0° or 90°, the inequalities in equation (5.2) may be further simplified to elicit *k*_2_, *β* > 0. No further explicit interrelationships between the model parameters or their numerical range may be directly elucidated from equation (5.2). However, this equation is used to check whether the parameters obtained by fitting the model in equation (3.4) to the experimental data satisfy the inequalities. Graphical representation of the condition of strict local convexity reflects itself in the convexity of the projections of the contours of constant *W* in the (*λ*_1_,*λ*_2_) and (*E*_11_,*E*_22_) planes, and will be presented in §6. For more in-depth analysis on conditions of convexity, the interested reader is referred to Holzapfel *et al*. [35] and Balzani *et al*. [36].

### 5.4. Fitting procedure

The model in equation (3.4) was fitted to the experimental data by the curve fitting toolbox in MATLAB^®^, using the Levenberg–Marquardt algorithm. The flowchart in figure 4 illustrates the different steps and sequences in estimating the model parameters, using the uniaxial stress–deformation curves in the transverse directions, obtained under different deformation rates. The first phase includes the estimation of the elastic parameters of the model, namely *α*, *β*, *k*_1_ and *k*_2_. In this phase, the elastic terms in *σ*_circumferential_ and *σ*_radial_ in equation (3.4) are fitted to the experimentally obtained elastic curves $\left(\dot{\lambda }=0.001\;s^{-1}\right)$ in each respective direction. As equation (3.2) indicates, parameters *α* and *β* are related to the ‘isotropic’ matrix, and shall therefore assume the same numerical values in both directions. With this consideration, the best fit in both the circumferential and radial directions is sought and the numerical values of the elastic parameters *α*, *β*, *k*_1_ and *k*_2_ are estimated. Once the parameters are verified to result in a convex *W*_e_, their numerical values are taken as the output of the fitting procedure for the elastic curves.

In the next phase, the values of *α*, *β*, *k*_1_ and *k*_2_ are incorporated into the model in equation (3.4) and are set fixed. The model is then fitted to the stress–deformation curves in circumferential and radial directions obtained under each considered deformation rate. As equation (3.3) indicates, *η*_1_ is related to the viscous properties of the matrix and shall therefore assume the same numerical value in both directions at each $\dot{\lambda }.$ With this consideration, the best fit in both the circumferential and radial directions is sought, and the numerical values of *η*_1_ and *η*_2_ at each $\dot{\lambda }$ are established. The convexity of the total energy function *W* is then verified graphically by plotting *W* and its contours in (*λ*_1_,*λ*_2_) and (*E*_11_,*E*_22_) planes (*E*_11_ and *E*_22_ represent the principal Green–Lagrange strains).

Taken together, this procedure enables quantification of the parameters of the transversely isotropic viscoelastic model in equation (3.4), describing the mechanical behaviour of the AV using uniaxial stress–deformation data in transverse directions.

## 6. Results

**Figure 5.**
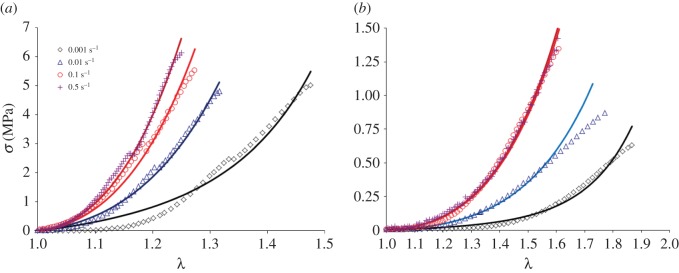
Fitting results for (*a*) circumferential and (*b*) radial loading directions. Hollow markers represent the experimental data and the continuous lines represent the best fit provided by the model.

Following the procedure described in §5, the model in equation (3.4) was fitted to the experimentally obtained *σ* − *λ* data. The model adequately captured the deformation behaviour of the specimens at each corresponding rate, reporting *R*^2^ values more than 0.97. Representative curves for both loading directions at each $\dot{\lambda }$ are presented in figure 5. The continuous line represents the model predictions.

The characterized model parameters are summarized in table 1, presented as mean ± s.e. Parameters *α*, *β*, *k*_1_ and *k*_2_ are the ‘elastic’ parameters and by definition are independent of the deformation rate; *η*_1_ and *η*_2_ are the parameters related to the viscous behaviour of the continuum, and therefore are rate-dependent. Note that *α* and *β* are the elastic parameters associated with the ‘isotropic’ matrix, and therefore are also independent of the direction of loading. These characteristics are reflected in the numerical values of the parameters listed in table 1.

**Table 1.** Model parameters at different deformation rates. Data are presented as mean ± s.e.

	*α* (MPa)	*β* (−)	*k*_1_ (MPa)	*k*_2_ (−)	*η*_1_ (MPa s^−1^)	*η*_2_ (MPa s^−1^)
radial direction						
elastic	0.0217 ± 0.005	1.389 ± 0.105	—	—	—	—
$\dot{\lambda }=0.01\;s^{-1}$	0.0217 ± 0.005	1.389 ± 0.105	—	—	6.082 ± 0.096	—
$\dot{\lambda }=0.1\;s^{-1}$	0.0217 ± 0.005	1.389 ± 0.105	—	—	1.821 ± 0.030	—
$\dot{\lambda }=0.5\;s^{-1}$	0.0217 ± 0.005	1.389 ± 0.105	—	—	0.3771 ± 0.002	—
circumferential direction						
elastic	0.0217 ± 0.005	1.389 ± 0.105	0.5853 ± 0.069	0.4250 ± 0.112	—	—
$\dot{\lambda }=0.01\;s^{-1}$	0.0217 ± 0.005	1.389 ± 0.105	0.5853 ± 0.069	0.4250 ± 0.112	6.082 ± 0.096	273.2 ± 7.70
$\dot{\lambda }=0.1\;s^{-1}$	0.0217 ± 0.005	1.389 ± 0.105	0.5853 ± 0.069	0.4250 ± 0.112	1.821 ± 0.030	58.75 ± 2.08
$\dot{\lambda }=0.5\;s^{-1}$	0.0217 ± 0.005	1.389 ± 0.105	0.5853 ± 0.069	0.4250 ± 0.112	0.3771 ± 0.002	16.68 ± 0.24

**Figure 6.**
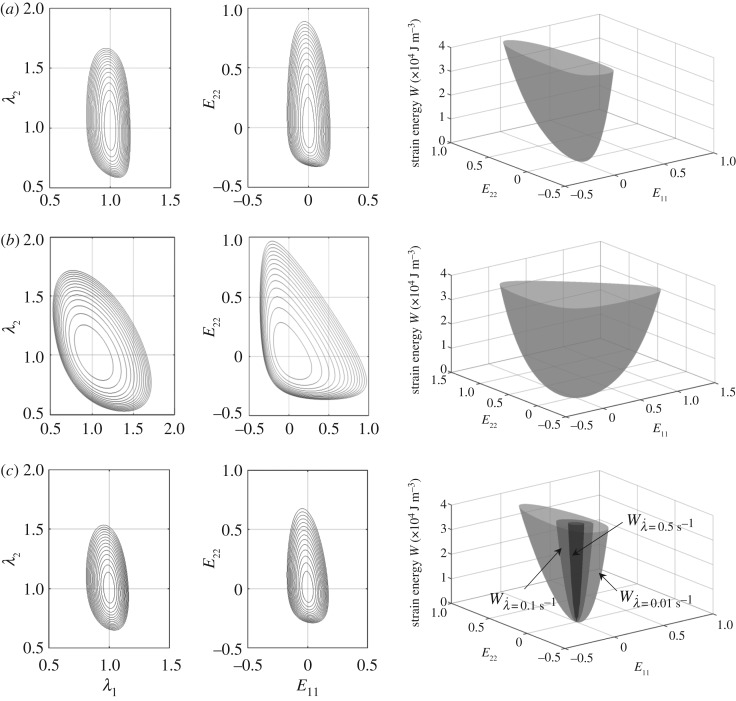
The energy function *W* and its contours in the (*λ*_1_,*λ*_2_) and (*E*_11_,*E*_22_) planes. Note that *E*_11_ and *E*_22_ represent the principal Green–Lagrange strains. (*a*) The elastic energy function *W*_e_ for the circumferential direction, (*b*) the elastic energy function *W*_e_ for the radial direction and (*c*) total energy functions *W* in the circumferential direction for $\dot{\lambda }=0.01\;s^{-1},$ $\dot{\lambda }=0.1\;s^{-1}$ and $\dot{\lambda }=0.5\;s^{-1}.$_._

The plots for *W*_e_ and its contours in the (*λ*_1_,*λ*_2_) and (*E*_11_,*E*_22_) planes, constructed using the values given in table 1, are shown in figure 6, for the circumferential (figure 6*a*) and the radial (figure 6*b*) directions, confirming the convexity of the elastic energy function. The numerical values of the model parameters listed in table 1 all report convex total energy functions. Figure 6*c* illustrates those energy functions in the circumferential loading direction. Contours of *W* in the circumferential direction for $\dot{\lambda }=0.01\;s^{-1}$ are also presented.

## 7. Discussion

A new transversely isotropic viscoelastic model was developed and presented in this paper to describe the behaviour of the AV tissue under uniaxial tensile deformation. The model accounts for the rate effects, by incorporating the rate of deformation $\dot{\lambda }$ as an explicit parameter. The rate effects were considered in the form of viscous damping *η*, formulated as a function of **C** and $\dot{\text{C}}$ invariants. We therefore note that the model is applicable to monotonic proportional loading conditions, i.e. tensile deformations, and may not be suitable for directly modelling relaxation or creep behaviours. While applied to uniaxial data, the model was developed within the general three-dimensional context, with the appropriate mathematical and mechanical conditions introduced at each step to tailor the model for application to uniaxial tensile data.

Embedded within the final form of the model in equation (3.4) are the assumptions of ‘thin sheet membrane’ and ‘pure homogenous deformation’. It is therefore important to note that equation (3.4) may not be suitable for application to situations where through-thickness or deformation under shear analyses are required. For further discussions on the scope of application of such continuum-based models, the interested reader is referred to the contributions made by Holzapfel and co-workers [20,21]. We further note that uniaxial tensile data alone are not sufficient for complete characterization of the multidimensional behaviour of the AV tissue.

As customary in studies modelling the biomechanical behaviour of soft tissues, model parameters and material properties are obtained in relation to experimental data, which itself is affected by the specimen samples and the experimental set-up. The AV tissue, similar to other biological soft tissues, is subject to sample variability. The deformation tests themselves are also affected by the properties of the experimental set-up such as the gripping mechanism, shape and size of the samples, and alignment of the gripped specimens, to name but a few. In particular, it has been previously shown that for radially cut specimens the gripping effects may compound the observed stress–strain behaviour of the samples [37,38], as the characteristic decay length may be the same order of magnitude as the gauge length of the samples (10 mm). Therefore, a degree of tolerance and caution has to be afforded to the numerical values of the model parameters reported in this study, and indeed in any such studies. Nonetheless, the detailed mathematical basis upon which our model was developed, together with the rigorous experimental campaign employed in this study, provides a solid basis for better understanding of the material properties of the AV and modelling its biomechanical behaviour.

Based on the modelling results summarized in table 1, a reduction is observed in the numerical values of *η_1_* and *η*_2_ with increase in $\dot{\lambda }.$ In rheological terms, this behaviour is referred to as a ‘shear-thinning’ behaviour. We recall that *η*_1_ and *η*_2_ are parameters associated with the dissipative effects of the viscous matrix and the fibre kinematics, respectively. The reduction in the values of *η* with $\dot{\lambda }$ in the AV has been observed and reported previously [2,6], and associated with shear-thinning behaviour of the GAG constituents of the valve. Therefore, the reduction in *η*_1_ values we have reported with an increase in $\dot{\lambda }$ may be interpreted as the reflection of the shear-thinning behaviour of the GAGs. The physical interpretation of the behaviour of *η*_2_, however, may be less obvious. As *η*_2_ is associated with the dissipative effects of fibre kinematics, e.g. fibre sliding or reorientation, the reduction in *η*_2_ values with increase in $\dot{\lambda }$ may stem from a decrease in these dissipative effects at higher deformation rates. At higher $\dot{\lambda },$ less time is afforded to the fibres to slide against each other, or to return back to their initial orientation during the deformation, compared with the case at lower $\dot{\lambda }.$ Further microstructural evidence concerning fibre kinematics within the AV tissue during deformation and relaxation/creep is required to reach a more concrete conclusion.

**Figure 7.**
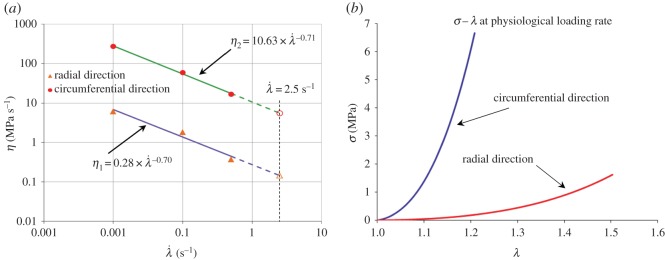
(*a*) The variation of *η*_1_ and *η*_2_ with $\dot{\lambda },$ and extrapolation to the physiological loading rate of $\dot{\lambda }=2.5\;s^{-1},$ plotted in logarithmic scale. The equations of the lines of best fit are $\eta_{1}=0.28\times {\dot{\lambda }}^{-0.70}$ and $\eta_{2}=10.63\times {\dot{\lambda }}^{-0.71};$ (*b*) the predicted *σ*−*λ* curves of the AV at physiological loading rates in both loading directions. The predicted curves were generated using the model in equation (3.4), and the extrapolated values of *η*_1_ = 0.15 MPa s^−1^ and *η*_2_ = 5.54 MPa s^−1^. The elastic parameters are listed in table 1.

To estimate the stress–strain behaviour of the AV tissue at physiological rates, where $\dot{\lambda }=2.5\;s^{-1}$ (corresponding to the physiological strain rate of 15 000% min^−1^ as reported in [16]), the values of *η*_1_ and *η*_2_ at that rate shall be extrapolated from the available data. The graph in figure 7*a* shows the variation of *η*_1_ and *η*_2_ with $\dot{\lambda },$ as given in table 1, in a logarithmic scale. Using an allometric function to fit to the (*η*,$\dot{\lambda }$) data points, the values of *η*_1_ and *η*_2_ at $\dot{\lambda }=2.5\;s^{-1}$ are calculated to be 0.15 and 5.54 MPa s^−1^, respectively (figure 7). Incorporating these values into equation (3.4), the predicted *σ* − *λ* curves of the AV tissue at physiological loading rates are illustrated in figure 7*b* for both loading directions. To the best of the authors' knowledge, this study presents the first prediction of *σ* − *λ* curves for the AV in the principal loading directions at physiological loading rates using a continuum-based model incorporating the deformation rate as an explicit variable. We note that some experimental data exist in the literature in relation to the peak stretches experienced by the AV *in vivo*, measured at the systolic and diastolic phases of the cardiac cycle [39,40]. Owing to experimental limitations, complete stress–strain curves were not established. Nonetheless, the reported values for stress at maximum diastolic stretches of approximately 1.13 in the circumferential direction (approx. 2.9 MPa) [39] correspond well with that from our predicated circumferential *σ* − *λ* curve (approx. 2.6 MPa; figure 7*b*). There is also some literature reporting membrane tension versus stretch curves for porcine AV specimens, obtained at rates close to physiological rates under equi-biaxial loading conditions *in vitro* [8]. However, these loading conditions appear to result in far larger circumferential and radial stretches than those measured *in vivo* [39,40], and drawing a direct relevance between those data and the mechanical behaviour of the AV at physiological rates, or our reported *σ* − *λ* curves, may be problematic.

**Figure 8.**
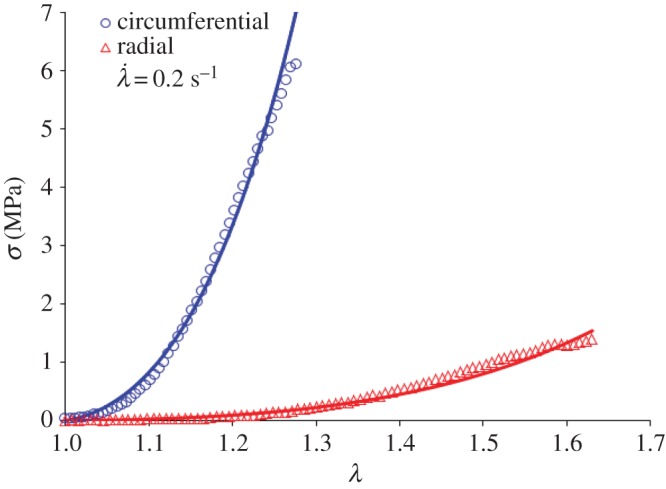
The representative *σ *−* λ* curve at $\dot{\lambda }=0.2\;s^{-1}.$ Hollow markers represent the experimental data, and the continuous lines represent the model predictions. The values for *η*_1_ and *η*_2_ to generate the model predictions were calculated using the line of best fit given in figure 7*a*, corresponding to 0.85 and 33.37 MPa s^−1^, respectively. The elastic parameters are listed in table 1.

To verify the reliability of the estimated values of *η*_1_ and *η*_2_, we used our newly established equation of the line of best fit to calculate the values of *η*_1_ and *η*_2_ at $\dot{\lambda }=0.2\;s^{-1}.$ Using these values, and the values for *α*, *β*, *k*_1_ and *k*_2_ in table 1, we used the model to predict the *σ* − *λ* curves at $\dot{\lambda }=0.2\;s^{-1}$ in both the circumferential and radial directions. We then performed tensile tests at that rate and obtained the corresponding *σ* − *λ* curves in both loading directions. The graphs in figure 8 illustrate the degree of conformity between the model predictions and the experimental data, reporting *R*^2^ values more than 0.99. Based on this result, the model predictions for physiological *σ* − *λ* curves may be treated with a high degree of confidence.

While the model presented in this study was primarily developed for application to the AV, the mechanical and mathematical criteria within which the model was derived are general and universal. Therefore, the model and the modelling approach presented here can be applied to other collagenous soft tissues with similar structural building blocks and a single preferred direction of the embedded collagen fibres, without the loss of generality.

## Data accessibility

Data for the reported *σ* − *λ* curves from a previous study [6] and the new datasets obtained for this study are available at University of Portsmouth research repository at: http://dx.doi.org/10.17029/754df175-2aa1-4859-993f-4cb775c7122d.
